# *Listeria*-Vectored Multiantigenic Tuberculosis Vaccine Enhances Protective Immunity against Aerosol Challenge with Virulent Mycobacterium tuberculosis in BCG-Immunized C57BL/6 and BALB/c Mice

**DOI:** 10.1128/mbio.00687-22

**Published:** 2022-06-01

**Authors:** Qingmei Jia, Saša Masleša-Galić, Susana Nava, Marcus A. Horwitz

**Affiliations:** a Division of Infectious Diseases, Department of Medicine, Center for Health Sciences, School of Medicine, University of California—Los Angeles, Los Angeles, California, USA; GSK Vaccines

**Keywords:** *Listeria monocytogenes*, *Listeria* vector, *Mycobacterium tuberculosis*, live vector vaccines, tuberculosis, tuberculosis vaccines

## Abstract

Mycobacterium tuberculosis infects approximately one-third of the world's population, causing active tuberculosis (TB) in ~10 million people and death in ~1.5 million people annually. A potent vaccine is needed to boost the level of immunity conferred by the current Mycobacterium bovis BCG vaccine that provides moderate protection against childhood TB but variable protection against adult pulmonary TB. Previously, we developed a recombinant attenuated Listeria monocytogenes (rLm)-vectored M. tuberculosis vaccine expressing the M. tuberculosis 30-kDa major secretory protein (r30/Ag85B), recombinant attenuated L. monocytogenes Δ*actA* ΔinlB prfA*30 (rLm30), and showed that boosting BCG-primed mice and guinea pigs with rLm30 enhances immunoprotection against challenge with aerosolized M. tuberculosis Erdman strain. To broaden the antigen repertoire and robustness of rLm30, we constructed 16 recombinant attenuated L. monocytogenes vaccine candidates expressing 3, 4, or 5 among 15 selected M. tuberculosis antigens, verified their protein expression, genetic stability, and growth kinetics in macrophages, and evaluated them for capacity to boost protective efficacy in BCG-primed mice. We found that boosting BCG-primed C57BL/6 and BALB/c mice with recombinant attenuated L. monocytogenes multiantigenic M. tuberculosis vaccines, especially the rLm5Ag(30) vaccine expressing a fusion protein of 23.5/Mpt64, TB10.4/EsxH, ESAT6/EsxA, CFP10/EsxB, and r30, enhances BCG-induced protective immunity against M. tuberculosis aerosol challenge. In immunogenicity studies, rLm5Ag(30) strongly boosts M. tuberculosis antigen-specific CD4-positive (CD4^+^) and CD8^+^ T cell-mediated TH1-type immune responses in the spleens and lungs of BCG-primed C57BL/6 mice but does so only weakly in BCG-primed BALB/c mice. Hence, rLm5Ag(30) boosts BCG-primed immunoprotection against M. tuberculosis aerosol challenge in both C57BL/6 and BALB/c mice despite major differences in the magnitude of the vaccine-induced Th1 response in these mouse strains. Given the consistency with which recombinant attenuated L. monocytogenes vaccines expressing the 5 M. tuberculosis antigens in rLm5Ag(30) are able to boost the already high level of protection conferred by BCG alone in two rigorous mouse models of pulmonary TB and the broad CD4^+^ and CD8^+^ T cell immunity induced by rLm5Ag(30), this vaccine holds considerable promise as a new vaccine to combat the TB pandemic, especially for the majority of the world’s population immunized with BCG in infancy.

## INTRODUCTION

Tuberculosis (TB), caused by Mycobacterium tuberculosis, remains a deadly global disease. It is estimated that one-quarter of the world’s population has been infected with M. tuberculosis, most of whom develop latent TB infection, and that 10 million people develop active TB and 1.5 million people die of TB annually. Mycobacterium bovis bacillus Calmette-Guérin (BCG), developed more than 100 years ago and the only licensed vaccine against TB, has been used to vaccinate infants and to protect young children against severe forms of TB; however, BCG has shown variable efficacy in preventing pulmonary TB in adolescents and adults, the most prevalent form (1). As BCG has been widely used worldwide to vaccinate 88% of infants within the first year of their life (1, 2), booster vaccines that improve upon the efficacy of BCG, even to a small extent, could have a significant impact on the TB pandemic.

Several strategies have been employed to develop replacement and booster vaccines for BCG against TB. BCG replacement vaccines include recombinant BCG (rBCG30) overexpressing the M. tuberculosis 30-kDa major secretory protein (r30/Ag85B) (3, 4), an iron-limited version of rBCG30 [rBCG(*mbtB*)30] with an inability to acquire iron but which can be preloaded with iron-siderophore and thereby multiply for several generations in the host (5), a recombinant BCG (BCG::Δ*ureC hly*^+^) engineered to acidify the phagosome and secrete listeriolysin (6), a live genetically attenuated M. tuberculosis vaccine (MTBVAC) (7); and killed whole-cell Mycobacterium vaccines, among others (8). BCG booster vaccines include primarily protein/adjuvant vaccines comprising fusion proteins of selected M. tuberculosis antigens administered with a strong T cell-stimulating adjuvant and viral-vectored vaccines, wherein viruses, including adenovirus, modified vaccinia Ankara virus, and cytomegalovirus, express recombinant proteins (9–15). Recent developments include substantial protection against M. tuberculosis challenge in rhesus macaques vaccinated intravenously with BCG (16), 50% protection against progression to pulmonary TB in humans vaccinated intramuscularly with a subunit vaccine (M72/ASO1_E_) (11), and 45% prevention of M. tuberculosis infection (defined as sustained conversion by QuantiFERON-TB Gold In-Tube assay) in adolescents revaccinated intradermally with BCG (12).

Previously, in our design of a novel TB booster vaccine, we employed a highly attenuated replicating bacterium as a vaccine vector, *Lm* Δ*actA* ΔinlB prfA*, a Listeria monocytogenes with deletions in two major virulence genes (*actA* and *inlB*) and a single amino acid substitution (G155S) in PrfA (positive regulatory factor A) resulting in constitutive overexpression of PrfA and PrfA-dependent genes, a modification exploited to enhanced vaccine efficacy (17). Listeria monocytogenes is an intracellular bacterium that invades mononuclear phagocytes, resides in a membrane-bound phagosome, and, ultimately, escapes the phagosome to reside and multiply in the cytoplasm (18). Its intraphagosomal and intracytoplasmic locations favor antigen presentation via both MHC class I and II, respectively, allowing induction of both CD4-positive (CD4^+^) and CD8^+^ antigen-specific T cells, both important to immunity against TB. A *Listeria* vector also has other immunologic advantages, including the capacity to carry and express a large amount of recombinant protein cargo; the ability to disseminate to organs that are impacted by M. tuberculosis, such as the lung and spleen, before being cleared by the immune system, thereby promoting local immunity at sites of M. tuberculosis infection; and the fact that preexisting immunity does not negatively affect efficacy (19, 20). Additional practical advantages of a *Listeria*-vectored vaccine are an established safety profile (21), as the vector has been used safely in cancer vaccines, and low cost of manufacture in simple broth culture without the need for extensive purification as in the case of protein/adjuvant and viral-vectored vaccines.

In a previous study, we developed a recombinant Listeria monocytogenes Δ*actA* ΔinlB prfA*-vectored vaccine candidate (rLm30) expressing the M. tuberculosis 30-kDa major secretory protein (r30/Ag85B/Rv1886) driven by the *hly* promoter and leader sequence or the *actA* promoter and leader sequence to facilitate the expression and secretion of r30 by recombinant attenuated L. monocytogenes. We found that rLm30 significantly enhances BCG-primed protective efficacy against aerosol challenge with the virulent M. tuberculosis Erdman strain in mice and guinea pigs (22). Boosting BCG-primed C57BL/6 mice with rLm30 induces strong antigen-specific T cell-mediated immune responses, including greater frequencies of antigen-specific polyfunctional CD4^+^ and CD8^+^ T cells expressing interferon gamma (IFN-γ), tumor necrosis factor alpha (TNF-α), and/or interleukin 2 (IL-2) in the spleens and lungs.

To expand the M. tuberculosis antigen repertoire of this recombinant attenuated L. monocytogenes vaccine, we evaluated 14 proteins in addition to r30 for potential inclusion in a multiantigenic vaccine. We constructed a total of 16 new recombinant attenuated L. monocytogenes multiantigenic vaccine candidates, including 11 vaccines expressing a fusion protein of 5 M. tuberculosis antigens, and evaluated them for efficacy against M. tuberculosis aerosol challenge in C57BL/6 and/or BALB/c mice. We identified rLm5Ag(30), expressing the fusion protein 23.5(Mpt64)-TB10.4(EsxH)-ESAT6(EsxA)-CFP10(EsxB)-r30(Ag85B), as one of the most promising vaccine candidates. We then studied the immunogenicity of rLm5Ag(30) in BCG-primed C57BL/6 and BALB/c mice. We found that while the recombinant attenuated L. monocytogenes-vectored multiantigenic vaccines boost BCG-primed protection in both C57BL/6 and BALB/c mice, the rLm5Ag(30) vaccine induces strong T cell-mediated immune responses, evidenced by enhanced antigen-specific frequencies of splenic and lung CD4^+^ and CD8^+^ T cells expressing IFN-γ, TNF-α, and/or IL-2, in BCG-primed C57BL/6 mice but not in BALB/c mice, where such responses are markedly limited. Thus, while the multiantigenic recombinant attenuated L. monocytogenes vaccines enhance protective immunity in both BCG-immunized mouse strains, they do so via disparate immune responses.

## RESULTS

### Construction and verification of new recombinant attenuated L. monocytogenes vaccines expressing 1, 3, or 4 recombinant M. tuberculosis proteins.

Previously we have shown that rLm30, expressing the r30/Ag85B downstream of the Lm *actA* promoter and ligated to the N-terminal 100 amino acids of ActA (ActAN) as a fusion protein, boosts BCG-primed efficacy against TB (22). To expand the M. tuberculosis antigen repertoire of the recombinant attenuated L. monocytogenes vaccine platform, we initially constructed 3 new recombinant attenuated L. monocytogenes vaccine candidates, rLm23.5, expressing the mature peptide of 23.5/Mpt64(Δ1V-23A); rLm3Ag, expressing the fusion protein of Ag85B(Δ2Q-43A)-RP-TB10.4-GGSG-ESAT6 (RP; a dipeptide encoded by EagI restriction enzyme site [*CGGCCG*] for cloning purposes; GGSG, a flexible fusion protein linker); and rLm4Ag, expressing the fusion protein of Mpt64(Δ1V-23A)-RP-TB10.4-GGSG-ESAT6-GSSGGSSG-CFP10 (GSSGGSSG, a flexible linker) (Table 1). The M. tuberculosis antigens in each vaccine construct were expressed as a C-terminal fusion protein to Lm ActAN; the M. tuberculosis protein expression cassette was driven by the Lm *actA* promoter and integrated at the *tRNA^arg^* locus of the *Lm* Δ*actA* ΔinlB prfA* chromosome.

**TABLE 1:** recombinant attenuated L. monocytogenes vaccine candidates

Vaccine	Antigen expression cassette	Estimated pI/MW^*a*^ (Da)	Integration locus
rLm23.5	ActAN-Mpt64(Δ1V-23A)	4.54/30,560	*tRNA^arg^*
rLm3Ag	ActAN-Ag85B(Δ2Q-43A)-TB10.4-ESAT6	4.65/59,691	*tRNA^arg^*
rLm4Ag	ActAN-Mpt64(Δ1V-23A)-TB10.4-ESAT6-CFP10	4.57/62,683	*tRNA^arg^*

a pI, isoelectric point; MW, molecular weight.

We verified the expression of the heterologous protein ActAN-Mpt64 by rLm23.5 as a 31-kDa protein band detected by a polyclonal antibody to a peptide comprising 18 amino acids (A30-K47) of ActAN (AK18) (courtesy of J. Skoble and P. Lauer) (see Fig. S1a in the supplemental material), expression of ActAN-Ag85B-TB10.4-ESAT6 by rLm3Ag as a 59-kDa protein band detected by a rabbit polyclonal antibody to r30 (Fig. S1b, top) or a polyclonal antibody to TB10.4 (Fig. S1b, bottom), and expression of ActAN-Mpt64-TB10.4-ESAT6-CFP10 by rLm4Ag as a 63-kDa protein band detected by AK18 (Fig. S1c).

10.1128/mbio.00687-22.1 FIG S1 Heterologous protein expression by recombinant attenuated L. monocytogenes vaccines expressing 1, 3, or 4 antigens. (a) Expression of rLm23.5 by recombinant attenuated L. monocytogenes grown on agar. Lysates of three single colonies of rLm23.5 expressing the M. tuberculosis 23.5/Mpt64 protein as a C-terminal fusion protein to ActAN were prepared and analyzed by Western blotting using a polyclonal antibody to a peptide comprising 18 amino acids (A30-K47) of ActAN (Ak18). The expected protein band (31 kDa) is indicated by an arrow to the right of the gel. (b) Expression of heterologous fusion protein by rLm3Ag grown on agar. Single colonies of rLm30-10.4-ESAT6 (rLm3Ag) vaccine candidates were grown on BHI agar overnight. Multiple individual colonies of each candidate were lysed in SDS buffer and analyzed by SDS-PAGE and Western blotting by using a polyclonal antibody to r30 (top). The membrane was stripped and reprobed with a polyclonal antibody to TB10.4 (bottom). The expected protein bands of the rLm30-10.4-ESAT6 in the top and bottom panels were estimated as 59.4 and 59.7 kDa, respectively, indicated by an arrow to the right of the gel. A 10-kDa protein band in the bottom panel was specifically recognized by the antibody to TB10.4, also indicated by an arrow to the right of the gel, suggesting a breakdown product of the fusion protein. The 30-kDa major secretory protein (r30) control was recognized only by polyclonal antibody to r30, overlapping with a nonspecific protein band (lane 2, top panel). Lane 1, protein standards; lane 2, r30 protein control; lanes 3 and 4, rLm30-10.4-ESAT6; lanes 5, 6, and 7, rLm30-10.4-GGSG-ESAT6. (c) Expression of heterologous fusion proteins by rLm4Ag in infected murine macrophage-like cells. Monolayers of J774A.1 cells were uninfected or infected at an MOI of 10 with the L. monocytogenes vector or rLm4Ag expressing the fusion protein of 23.5-10.4-ESAT6-CFP10 that had been grown to stationary phase at 30°C. At 5.5 h postinfection, the infected cells were lysed and processed by Western blotting using a polyclonal antibody to ActA AK18. Culture filtrate proteins of rLm23.5-10.4-ESAT6-CFP10 were prepared and used as controls. The expected protein band (63 kDa) is indicated by an arrow to the right of the gel. Lane 1, protein standards; lane 2, lysate of uninfected J774A.1 cells; lane 3, culture filtrate of L. monocytogenes vector grown in broth culture; lane 4, lysate of J774A.1 cells infected with rLm23.5-10.4-ESAT6-CFP10; lane 5, culture filtrate proteins of rLm4Ag (23.5-10.4-ESAT6-CFP10). The molecular weights (MW) of protein standards are labeled to the left of each gel. Download FIG S1, EPS file, 2.4 MB. Copyright © 2022 Jia et al. 2022 Jia et al. https://creativecommons.org/licenses/by/4.0/ This content is distributed under the terms of the Creative Commons Attribution 4.0 International license.

### Boosting BCG-primed C57BL/6 mice with the combined rLm3Ag and rLm23.5 vaccines enhances protection against aerosolized M. tuberculosis.

**FIG 1:**
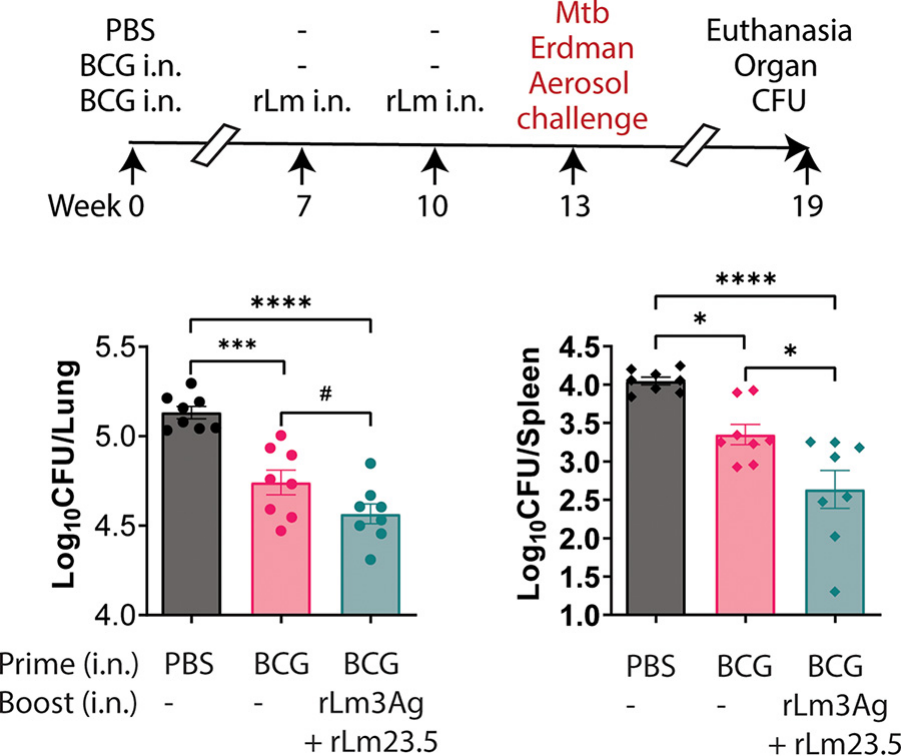
Efficacy against M. tuberculosis aerosol challenge of boosting BCG-primed mice with a combination of recombinant attenuated L. monocytogenes vaccines expressing 4 M. tuberculosis antigens. Female C57BL/6 mice (*n* = 8/group) were immunized intranasally (i.n.) with PBS (sham) or BCG at week 0; not boosted or boosted i.n. twice with 10^6^ CFU total of combined recombinant attenuated L. monocytogenes expressing 4 M. tuberculosis proteins, rLm3Ag (expressing Ag85B, TB10.4, and ESAT6) and rLm23.5 at weeks 7 and 10; challenged with aerosolized M. tuberculosis at week 13; and euthanized at week 19 (top). Lungs (bottom left) and spleens (bottom right) of mice were assayed for organ bacterial burden. One symbol represents one animal. Values are mean log_10_ CFU ± SEM. *, *P < *0.05; ***, *P < *0.001; ****, *P < *0.0001 by one-way ANOVA with Tukey’s multiple-comparison test (Prism v9.2.0); #, *P < *0.05, by one-way ANOVA and Fisher’s LSD criteria (Prism v9.2.0).

To determine the efficacy of these multiantigenic vaccine candidates as a booster vaccine in protecting BCG-immunized mice against aerosolized M. tuberculosis challenge, we immunized C57BL/6 mice, 8/group, intranasally (i.n.) with phosphate-buffered saline (PBS) (sham) or BCG at week 0 and boosted one group of BCG-immunized mice i.n. with a combination of recombinant attenuated L. monocytogenes vaccines expressing 4 M. tuberculosis antigens, rLm3Ag (expressing Ag85B-10.4-ESAT6) plus rLm23.5 at weeks 7 and 10. The mice were then challenged with aerosolized M. tuberculosis Erdman (2.6 × 10^5^ CFU for 30 min, resulting in an average of 21 CFU in the lungs at day 1 postchallenge) at week 13, euthanized at week 19, and their lungs and spleens assayed for M. tuberculosis CFU. As shown in Fig. 1, mice primed-boosted with BCG with rLm3Ag and rLm23.5 had a significantly lower bacterial burden in their lungs and spleens than sham-immunized mice and mice immunized with BCG. This result indicates that boosting BCG-primed C57BL/6 mice with an recombinant attenuated L. monocytogenes multiantigenic vaccine enhances immunoprotection against M. tuberculosis aerosol challenge.

### Boosting BCG-primed BALB/c mice with the combined rLm4Ag and rLm30 vaccines enhances protection against aerosolized M. tuberculosis.

**FIG 2:**
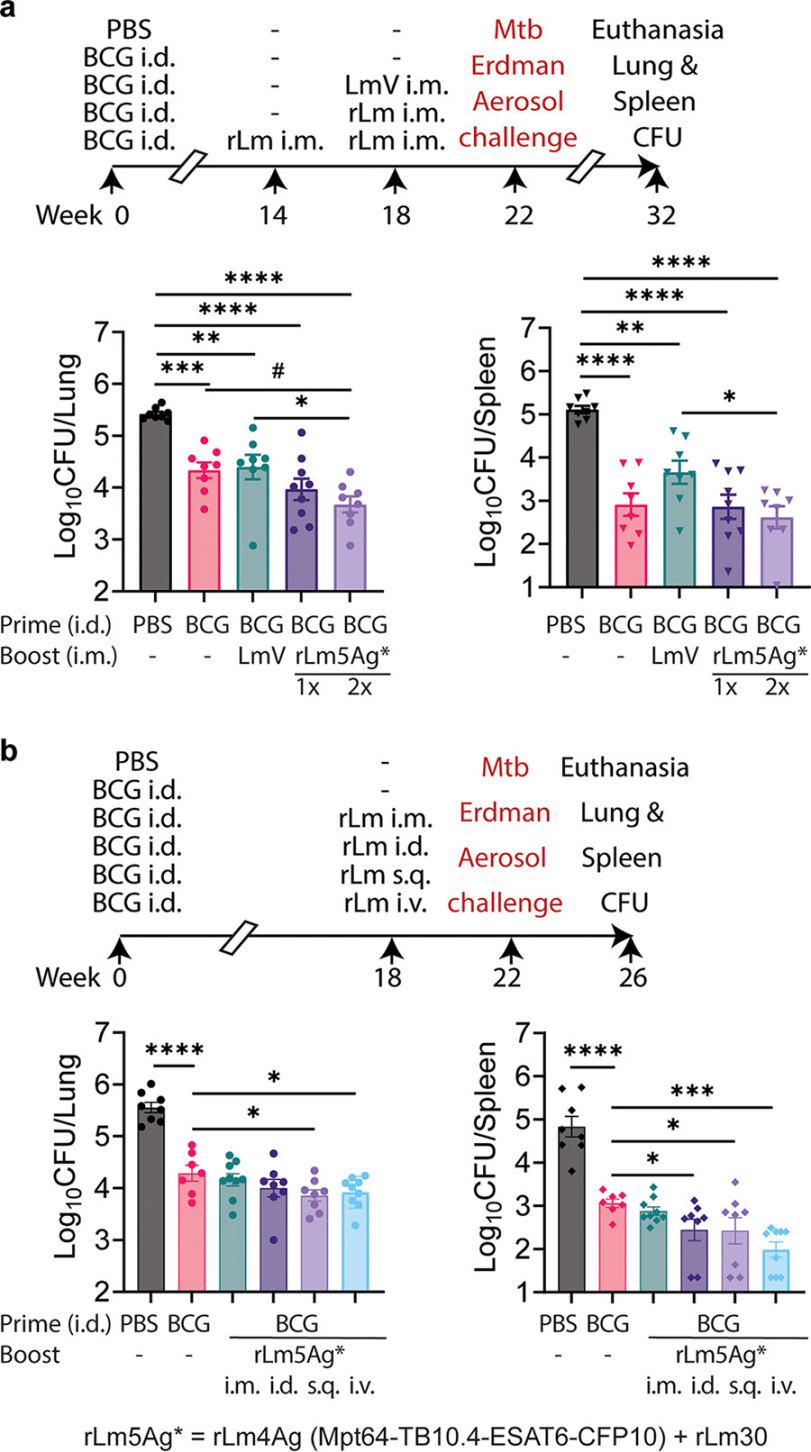
Efficacy against M. tuberculosis aerosol challenge of boosting BCG-primed mice with a combination of recombinant attenuated L. monocytogenes vaccines expressing 5 M. tuberculosis antigens. (a) Efficacy of boosting with 5Ag recombinant attenuated L. monocytogenes vaccines i.m. once versus twice. BALB/c mice (*n* = 8/group) were immunized i.d. with PBS or with BCG at week 0. BCG-primed mice were either not boosted or boosted intramuscularly (i.m.) or subcutaneously (s.c.) once (×1) at week 18 with L. monocytogenes vector (LmV) or the combination of rLm4Ag (expressing M. tuberculosis fusion protein Mpt64-TB10.4-ESAT6-CFP10) and rLm30. An additional group was boosted twice (×2) at weeks 14 and 18 with rLm4Ag plus rLm30. The mice were then challenged with aerosolized M. tuberculosis Erdman (average of 19 CFU delivered to the lungs of each animal, as assayed at day 1 postchallenge to 2 mice) at week 22 and euthanized at week 32 (top). Lungs (bottom left) and spleens (bottom right) of mice were assayed for organ bacterial burden. Shown are means ± SEM. One symbol represents one animal. Organ log_10_ CFU were analyzed by one-way ANOVA. *, *P < *0.05; ****, *P < *0.0001 by Tukey’s multiple-comparison test; #, *P < *0.05 by Fisher’s LSD test (Prism 9.2.0). (b) Efficacy of 5Ag recombinant attenuated L. monocytogenes vaccines by route of administration. BALB/c mice (8/group) were immunized i.d. with PBS (sham) or 5 × 10^5^ CFU BCG at week 0. Mice immunized i.d. with BCG were either not boosted or boosted i.m., i.d., subcutaneously (s.c.), or intravenously (i.v.) once at week 18 with 2 × 10^6^ CFU of the combination of rLm4Ag (expressing Mpt64-TB10.4-ESAT6-CFP10) and rLm30. The mice were then challenged at week 22 with aerosolized M. tuberculosis Erdman at the same time as the companion experiment shown in panel a (average of 19 CFU delivered to the lungs of each animal) and euthanized at week 26 (top). Afterward, lungs (bottom left) and spleens (bottom right) were removed and assayed for bacillus burdens. Shown are means ± SEM. Each symbol represents one mouse. *, *P < *0.05; ***, *P < *0.001; ****, *P < *0.0001 by one-way ANOVA with Fisher’s LSD test (Prism 9.2.0).

To further verify the immunoprotection against M. tuberculosis challenge of multiantigenic recombinant attenuated L. monocytogenes vaccine candidates as a booster vaccine in BCG-immunized mice, we immunized and challenged a different strain of mice, BALB/c mice. We immunized BALB/c mice, 8/group, intradermally (i.d.) with PBS (sham) or BCG at week 0 and did not boost or boosted BCG-immunized mice intramuscularly (i.m.) with L. monocytogenes vector or with a combination of recombinant attenuated L. monocytogenes vaccines now expressing 5 M. tuberculosis antigens—rLm4Ag (expressing Mpt64-TB10.4-ESAT6-CFP10) with rLm30 (the combined vaccines are abbreviated as rLm5Ag*)—at weeks 14 and 18. The mice were then challenged with aerosolized M. tuberculosis Erdman (2.6 × 10^5^ CFU for 30 min, resulting in an average of 19 CFU in the lungs at day 1 postchallenge) at week 22, euthanized at week 32, and their lungs and spleens assayed for M. tuberculosis CFU (Fig. 2a, top). As shown in Fig. 2a, bottom panels, immunizing with BCG alone was highly effective, reducing CFU in the lung band spleen by 1.1- and 2.2-log_10_ CFU, respectively, compared with sham immunization. Mice primed-boosted with BCG i.d.-rLm5Ag* i.m. once or twice had significantly lower log_10_ CFU in their lungs (*P < *0.0001) and spleens (*P < *0.0001) than the sham-immunized mice; in mice boosted twice, CFU in the lungs were 1.75-log_10_ CFU lower and, in the spleens, 2.5 log_10_ lower than in sham-immunized animals, thus attaining a ≥1.75-log_10_ CFU threshold of protection achieved by only a small minority of TB booster vaccines administered to BCG-immunized animals (23). Of note, mice primed with BCG i.d. and boosted with rLm5Ag* i.m. twice had significantly lower CFU in their lungs than mice immunized i.d. with BCG alone (*P < *0.05) or primed with BCG and immunized with L. monocytogenes vector (*P < *0.05) despite the high level of protection conferred by BCG alone. These mice also had significantly fewer CFU in their spleen than BCG-immunized mice boosted with the L. monocytogenes vector (*P < *0.05) despite the high level of protection (1.5-log_10_ CFU) conferred by the BCG-L. monocytogenes vector prime-boost. These results verify in BALB/c mice that boosting BCG-primed mice with an recombinant attenuated L. monocytogenes multiantigenic vaccine enhances immunoprotection against M. tuberculosis aerosol challenge.

In a companion experiment challenged at the same time as the experiment described in Fig. 2a, we compared different delivery routes for the recombinant attenuated L. monocytogenes multiantigenic booster vaccines. We immunized BALB/c mice, 8/group, i.d. with 5 × 10^5^ CFU of BCG at week 0 and boosted them once at week 18 with rLm5Ag* (a combination of recombinant attenuated L. monocytogenes Mpt64-TB10.4-ESAT6-CFP10 and rLm30) via the i.d., intravenous (i.v.), subcutaneous (s.c.), or intramuscular (i.m.) route. Mice immunized i.d. with PBS (Sham) or BCG at week 0 and not boosted served as controls. At week 22, we challenged the mice with aerosolized M. tuberculosis Erdman (as described in the companion experiment above). At week 26, we euthanized the mice and assayed bacillus burdens in their lungs and spleens (Fig. 2b, top).

As shown in Fig. 2b, bottom panels, immunizing with BCG i.d. alone was highly effective in comparison with sham immunization, reducing CFU by 1.3 log_10_ in the lung and 1.8 log_10_ in the spleen. Despite the especially high efficacy of BCG alone, BCG prime recombinant attenuated L. monocytogenes boosting further reduced CFU in the lungs and spleens after aerosol challenge with M. tuberculosis; the differences in CFU between boosted and nonboosted mice were statistically significant in the lungs for boosting by the s.c. (*P < *0.05) or i.v. route (*P < *0.05) and in the spleens for boosting via the s.c. route (*P < *0.05), i.d. route (*P < *0.05), or i.v. route (*P < *0.001). Of note, boosting BCG-primed mice via the i.d., s.c., or i.v. routes reduced CFU compared with sham-immunized mice by 1.6- to 1.7-log_10_ CFU in the lungs and 2.4- to 2.8-log_10_ CFU in the spleen.

Among the 4 boosting routes tested, the effectiveness in reducing lung CFU by route was s.c. > i.v. > i.d. > i.m., lowering log_10_ CFU by 0.43, 0.37, 0.29, and 0.13 log, respectively, versus BCG alone, and the effectiveness in reducing spleen CFU by route was i.v. > s.c. > i.d. > i.m., lowering log_10_ CFU by 1.07, 0.63, 0.61, and 0.18 log, respectively, versus BCG alone. Thus, the most effective routes were s.c. and i.v., and s.c. was the superior route in the lung. Surprisingly, the i.m. route was the least efficacious in both the lung and spleen. However, in the companion experiment described above (Fig. 2a), boosting i.m. twice with rLm5Ag* (rLm4Ag with rLm30) provided improved protection in the lung (*P < *0.05) and spleen compared with boosting once. Given the safety and practical advantage of the s.c. route of administration and the fact that the s.c. route was superior to other routes in the lung, the major site of M. tuberculosis pathology, we selected the s.c. route for future studies.

### Selection of M. tuberculosis antigens and construction of 13 new rLm5Ag vaccine candidates.

To further expand the M. tuberculosis antigen repertoire, we selected 15 M. tuberculosis proteins (including r30) as potential vaccine candidates for further investigation (Table 2), including (i) secreted proteins r30 (4), Mpt64 (24–26), TB8.4 (27), and Apa (28); (ii) ESAT6 and associated proteins secreted by the Esx/type VII secretion system, ESAT6 (24, 29), CFP10 (30), TB10.4 (31, 32), EspA (24), EspC (24), and EsxN; (iii) antigenic PE/PPE proteins PE25 (33) and PPE68 (24, 34); and (iv) latency-associated proteins α-crystallin/hspX (35, 36), Hrp1 (37), and VapB47 (37, 38). Among the 15 selected proteins, all but two (EsxN and PE25) have been shown by us or others to be immunoprotective antigens when incorporated into various vaccines, including protein/adjuvant, DNA, *Listeria*-vectored, or virus-vectored vaccines, and 4 proteins, Mpt64, PPE68, CFP-10, and ESAT-6, are absent either from all BCG strains or the modern BCG strain (Mpt64) (39, 40) (Table 2).

**TABLE 2:** Fifteen M. tuberculosis proteins selected as vaccine candidates

Gene Rv no.	Product (62)	Length (aa)	Protection in animal models^*a*^ (reference[s])	Absence in BCG	Homolog in BCG^*b*^^,^^*c*^
0288	TB10.4/EsxH/CFP-7, ESAT6 family	96	31	No	0328
1174c	TB8.4, low-molecular-wt T-cell antigen	110	27	No	1237c
1793	EsxN, putative ESAT-6-like protein	94		No	1825
1860	Apa, alanine and proline rich secreted glycoprotein	325	28	No	1896
1886c	r30/Ag85B/FbpB, 30-kDa major secreted protein	325	4, 9, 41	No	1923c
1980c	23.5/antigen Mpt64	228	25, 26	Yes	
2031	HspX, heat shock protein HspX (α-crystallin homolog)	144	35, 36	No	2050c
2431c	PE25, Esx-5, secreted with PPE41 and EspG5	99		No	2450c
2626c	HrpI, hypoxic response protein 1	143	37	No	2653c
3407	Antitoxin VapB47, part of the toxin-antitoxin operon with Rv3408	99	37, 38	No	3477
3615c	EspC, ESX-1 secretion-associated protein C	103	24	No	3679c
3616c	EspA, ESX-1 secretion-associated protein A	392	24	No	3680c
3873	PPE68, interacts with ESAT6, CFP10, and TB10.4	368	24	Yes	
3874	CFP-10/EsxB, cotranscribed with Rv3875	100	30	Yes	
3875	ESAT-6/EsxA, early secretory antigen target	95	24, 29	Yes	

a As an individual protein.

b Blasted using nucleotide BLAST tool via https://blast.ncbi.nlm.nih.gov/Blast.cgi.

c M. bovis BCG reference strain, Pasteur 1173P2, GenBank accession number AM408590.1.

We constructed 11 new rLm5Ag vaccine candidates carrying a single copy of an ActAN-M. tuberculosis 5-antigen fusion protein expression cassette downstream of the Lm *actA* promoter integrated at the 3′ end of the *tRNA^arg^* locus in the recombinant attenuated L. monocytogenes chromosome; in all such cases, the first four proteins in the fusion protein were Mpt64-EsxH-EsxA-EsxB followed by a GSSGGSSG flexible linker, and the fifth protein was 1 of the 11 other selected M. tuberculosis proteins in Table 2. In addition, we constructed 2 recombinant attenuated L. monocytogenes vaccine candidates, rLm4Ag(*comK*) expressing the M. tuberculosis 4Ag (Mpt64-EsxH-EsxA-EsxB) fusion protein, and rLm30(*comK*) expressing Ag85B from the *comK* locus of the recombinant attenuated L. monocytogenes chromosome, respectively, to allow a comparison of vaccines expressing proteins at this locus versus the *tRNA^arg^* locus and to explore the possibility of later expressing proteins from both loci in the same vaccine (Table 3).

**TABLE 3:** Thirteen new recombinant attenuated L. monocytogenes multiantigenic vaccine candidates

Vaccine	M. tuberculosis antigen expression cassette^*a*^	Estimated pI/MW (Da)^*b*^	Integration locus
rLm5Ag(30)	ActAN-Mpt64-EsxH-EsxA-EsxB-r30(Δ2Q-43A)	4.63/94,106	*tRNA^arg^*
rLm5Ag(EspA)	ActAN-Mpt64-EsxH-EsxA-EsxB-EspA(Δ111F-193L)	4.69/94,889	*tRNA^arg^*
rLm5Ag(EspC)	ActAN-Mpt64-EsxH-EsxA-EsxB-EspC	4.64/74,239	*tRNA^arg^*
rLm5Ag(EsxN)	ActAN-Mpt64-EsxH-EsxA-EsxB-EsxN	4.59/73,386	*tRNA^arg^*
rLM5Ag(PPE68)	ActAN-Mpt64-EsxH-EsxA-EsxB-PPE68	4.46/100,774	*tRNA^arg^*
rLm5Ag(PE25)	ActAN-Mpt64-EsxH-EsxA-EsxB-PE25(Δ66I-73L)	4.67/73,422	*tRNA^arg^*
rLm5Ag(Apa)	ActAN-Mpt64-EsxH-EsxA-EsxB-APA(Δ2H-39A)	4.55/92,355	*tRNA^arg^*
rLm5Ag(HspX)	ActAN-Mpt64-EsxH-EsxA-EsxB-HspX(Δ121I-126V)	4.66/78,888	*tRNA^arg^*
rLm5Ag(TB8.4)	ActAN-Mpt64-EsxH-EsxA-EsxB-TB8.4(Δ2R-28A)	4.55/71,912	*tRNA^arg^*
rLm5Ag(VapB47)	ActAN-Mpt64-EsxH-EsxA-EsxB-VapB47	4.80/74,453	*tRNA^arg^*
rLm5Ag(Hrp1)	ActAN-Mpt64-EsxH-EsxA-EsxB-Hrp1	4.65/78,962	*tRNA^arg^*
rLm4Ag(*comK*)	ActAN-Mpt64-EsxH-EsxA-EsxB	4.57/62,683	*comK*
rLm30(*comK*)	ActAN-Ag85B(Δ2Q-43A)	4.67/38,789	*comK*

a Mpt64 refers to Mpt64(Δ1V-23A).

b The estimated *M*_w_ of each fusion protein is calculated as a secreted form without the signal peptide for ActA (1V-29A, 3.3 kDa).

**FIG 3:**
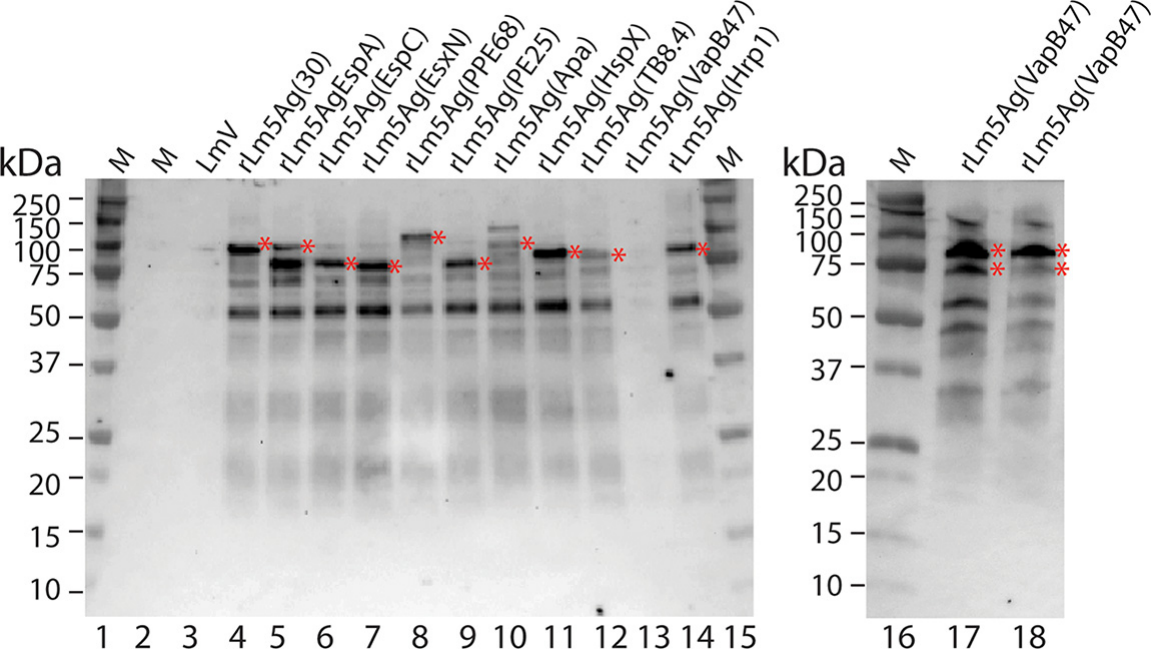
Expression of fusion proteins comprising 5 M. tuberculosis antigens by 11 new recombinant attenuated L. monocytogenes vaccine candidates grown in broth. Glycerol stocks of the L. monocytogenes vector and each of 11 new recombinant attenuated L. monocytogenes vaccine candidates expressing 5 M. tuberculosis antigens were grown in brain heart infusion medium supplemented with streptomycin (200 μg/mL) overnight at 37°C. Cells were collected by centrifugation and lysed in SDS buffer, and the cell lysate was processed by standard SDS-PAGE and Western blotting using a polyclonal antibody to ActA AK18. In both panels, the expected major protein bands are indicated by red asterisks to the right of each protein band. The MWs of the protein standards are labeled on the left of each panel. The M. tuberculosis fusion protein expression cassette and the estimated MW of the fusion protein for each of the vaccines listed at the top of the gels are described in Table 3. Note that lane 13 shows one clone, and lanes 17 and 18 show two different clones of the same construct, expressing the secreted (74-kDa) and nonsecreted (78-kDa) fusion proteins.

We examined M. tuberculosis fusion protein expression by rLm5Ag vaccine candidates grown in broth medium. As shown in Fig. 3, one major protein band (indicated by red asterisks to the right of the protein band) at the estimated molecular weight (MW) (Table 3, 3rd column from the left) of the fusion protein and multiple minor protein bands (possible N-terminal protein breakdown products) were detected in the lysates of 10 recombinant attenuated L. monocytogenes vaccine candidates (lanes 4 to 12 and 14) by the rabbit Ak18 polyclonal antibody to ActAN. No specific protein band was detected from the lysate of the L. monocytogenes vector (LmV, lane 3) as expected. One recombinant attenuated L. monocytogenes vaccine candidate (lane 13), rLm5Ag(VapB47), also showed no protein expression in the lysate; however, two other clones of the same construct did express a protein of the approximately expected MW (lanes 17 and 18). Similar protein bands were detected by the ActA AK18 antibody in the lysates of J774A.1 cells infected with the relevant recombinant attenuated L. monocytogenes vaccine candidates (data not shown). We also detected the M. tuberculosis protein expression by rLm4Ag(*comK*) and rLm30(*comK*) grown in broth medium (data not shown). Thus, we have verified that 13 new recombinant attenuated L. monocytogenes vaccine candidates express a fusion protein of the expected size when grown in broth and macrophages.

We also verified the M. tuberculosis protein expression cassette integrated at the *tRNA^arg^* locus by PCR and nucleotide sequencing of the resultant PCR products. As shown in Fig. S2a, amplification across the bacterial attachment site *tRNA^arg^-attBB′* with primers NC16 and PL95 resulted in a 548-bp fragment in each selected clone of the 10 recombinant attenuated L. monocytogenes candidates (lanes 3 to 10, 12, and 14); with regard to rLm5Ag(VapB47), 2 out of 3 clones selected tested positive (lanes 13, 15, and 16). Amplification with primers 319 and 327 across the antigen expression cassette resulted in various sizes of the PCR product, as shown in Fig. S2b. Consistent with the PCR result using primers NC16 and PL95, an ~2,154-bp DNA fragment was amplified with primers 319 and 327 from 2 out of 3 selected clones of rLm5Ag(VapB47) (Fig. S2b, lanes 13, 17, and 18).

10.1128/mbio.00687-22.2 FIG S2 Verification of recombinant attenuated L. monocytogenes vaccine candidates by PCR. Cells of L. monocytogenes vector or rLm5Ag vaccine candidates were collected from overnight culture in BHI broth medium, resuspended in water, and boiled to lyse the cells. Total cell lysates were subjected to PCR using primers NC16 and PL95 to amplify across the bacterial attachment site *tRNAarg-attBB′* amplifying a unified 548-bp fragment (a) and by using primers 319 and 327 to amplify across the integrated antigen expression cassette, amplifying fragments with various sizes, as indicated in the 3rd column from the left of the bottom panel (b). The sample loading orders are listed at the bottom of the left (a) and right (b) panels, respectively. The sizes of DNA standards are listed to the left of the gels, and the expected DNA fragments are indicated by arrows to the right of the gels. Download FIG S2, EPS file, 2.2 MB. Copyright © 2022 Jia et al. 2022 Jia et al. https://creativecommons.org/licenses/by/4.0/ This content is distributed under the terms of the Creative Commons Attribution 4.0 International license.

We tested the genetic stability of the pPL2e-vectored M. tuberculosis 5Ag expression cassette integrated at the *tRNA^arg^* locus of the recombinant attenuated L. monocytogenes chromosome by culturing the vaccine candidates in the presence and absence of erythromycin, a marker used for selection of the recombinant attenuated L. monocytogenes constructs. We observed that the M. tuberculosis 5Ag antigen expression cassettes were stable after passage *in vitro* in the absence of antibiotic selection, except for one clone of the rLm5Ag(VapB47) expressing Rv3407 as the fifth protein (Fig. S3). Rv3407 encodes an antitoxin virulence-associated protein, B47, that is part of the toxin-antitoxin operon with Rv3408. Expressing Rv3407 independently of Rv3408 may have resulted in instability of this recombinant attenuated L. monocytogenes fusion protein. However, when we grew the vaccine candidates carrying the antigen expression cassette, including Rv3407 in the presence of antibiotic selection, these vaccines expressed the M. tuberculosis fusion proteins abundantly with two major bands of ~74 (secreted form) and ~78 kDa (nonsecreted form) (Fig. 3, lanes 17 and 18).

10.1128/mbio.00687-22.3 FIG S3 The antigen expression cassettes in the rLm5Ag chromosomes are generally stable in the presence or absence of antibiotic selection. The L. monocytogenes vector or rLm5Ag vaccine candidates, as indicated below the horizontal axis, were inoculated into brain heart infusion (BHI) medium supplemented with streptomycin (200 μg/mL) (Strep) (the L. monocytogenes vector is streptomycin resistant) or streptomycin (200 μg/mL) plus erythromycin (2.5 μg/mL) (Strep+Erm2.5). The cultures were grown overnight at 37°C with 5% CO_2_, and optical density was measured at 540 nm (OD_540_). Download FIG S3, EPS file, 1.5 MB. Copyright © 2022 Jia et al. 2022 Jia et al. https://creativecommons.org/licenses/by/4.0/ This content is distributed under the terms of the Creative Commons Attribution 4.0 International license.

### Growth kinetics of rLm5Ag vaccine candidates in broth medium and in infected murine and human macrophages.

We examined the growth kinetics in brain heart infusion (BHI) broth of the 11 new rLm5Ag vaccine candidates. As shown in Fig. S4a to c, all of the rLm5Ag vaccine candidates, except rLm5Ag(VapB47) (this clone was subsequently discarded and replaced by a new clone shown in Fig. 3, lane 17), grew similarly in broth to the L. monocytogenes vector. rLm4Ag(comK) and rLm30(comK) also grew similarly to the L. monocytogenes vector in broth medium (data not shown).

10.1128/mbio.00687-22.4 FIG S4 Growth kinetics of recombinant attenuated L. monocytogenes vaccine candidates expressing fusion proteins of 5 M. tuberculosis antigens in broth and in murine and human macrophage-like cells. (a to c) Glycerol stocks of the L. monocytogenes vector and 11 recombinant attenuated L. monocytogenes vaccine candidates expressing M. tuberculosis 4Ag plus 1 of 11 M. tuberculosis antigens, as indicated below the bottom panel of each column of panels. (a) r30 or ESAT6-associated proteins EspA, EspC, or EspN (a); (b) PPE68, PE25, glycoprotein Apa, or low-molecular-weight T-cell antigen TB8.4; and (c) heat shock protein (HspX), antitoxin (VapB47), or hypoxic response protein (Hrp1) (c) were inoculated into brain heart infusion (BHI) medium supplemented with streptomycin (200 μg/mL) (the L. monocytogenes vector is streptomycin resistant) to prevent any contamination and grown overnight under stationary conditions in a 37°C incubator with 5% CO_2_. The overnight culture was inoculated into 5 mL fresh BHI with streptomycin with an initial optical density at 540 nm (OD_540_) of ~0.05; the subculture was incubated at 37°C with shaking at 180 rpm. At 0, 3, 5, and 7 h postinoculation, a 1-mL aliquot of each culture was removed, and the OD_540_ was measured. The OD_540_ of each recombinant attenuated L. monocytogenes vaccine strain was compared with that of the L. monocytogenes vector using 2-way ANOVA with Tukey’s multiple-comparison tests. *, *P < *0.05 between a recombinant attenuated L. monocytogenes vaccine and the L. monocytogenes vector. The experiments were repeated three times with similar results. (d to i) The overnight stationary culture was used to infect monolayers of murine macrophage cells (J774A.1) (d to f) or monolayers of human monocytic cells (THP-1) differentiated with PMA (g to i) at a multiplicity of infection of 10 for 90 min in DMEM (J774A.1) or RPMI (THP-1) medium supplemented with 10% heat-inactivated fetal bovine serum (HI-FBS). After 90 min infection, cells were washed three times with PBS supplemented with 2% HI-FBS. The infected cells were cultured for an additional 4.5 h in DMEM or RPMI medium supplemented with 10% HI-FBS and gentamycin (10 μg/mL). At 2, 4, and 6 h postinfection, medium was removed, cells were lysed in 0.1% saponin-PBS, cell lysates were serially diluted in PBS, and the diluted cell lysates were plated on BHI plus streptomycin (200 μg/mL) agar plates. The plates were incubated at 37°C for 2 days, and colonies were enumerated. The initial CFU count of the medium of each strain used for infection of cells is indicated at 0-hour time point. The CFU count of each strain at each time point is compared with that of the L. monocytogenes vector by 2-way ANOVA with Dunnett’s multiple-comparison test (Prism 7.04). *P* values are shown as asterisk(s) color-coded to various strains. *, *P < *0.05; **, *P < *0.01; ***, *P < *. 001. Download FIG S4, EPS file, 1.9 MB. Copyright © 2022 Jia et al. 2022 Jia et al. https://creativecommons.org/licenses/by/4.0/ This content is distributed under the terms of the Creative Commons Attribution 4.0 International license.

To examine the growth kinetics of rLm5Ag vaccine candidates in macrophage-like cells, we infected monolayers of murine J774A.1 cells or monolayers of human THP-1 cells differentiated by phorbol 12-myristate 13-acetate (PMA) with the L. monocytogenes vector or with the recombinant attenuated L. monocytogenes vaccine candidates at a multiplicity of infection (MOI) of 10. In general, rLm5Ag vaccine candidates expressing fusion proteins comprising M. tuberculosis 4Ag (Mpt64-TB10.4-ESAT6-CFP10) ligated with a 5th antigen grew similarly to the L. monocytogenes vector in both murine (Fig. S4d to f) and human (Fig. S4g to i) macrophage-like cells.

### Protective immunity of rLm5Ag vaccine candidates against aerosol challenge with virulent M. tuberculosis Erdman strain in BALB/c mice.

**FIG 4:**
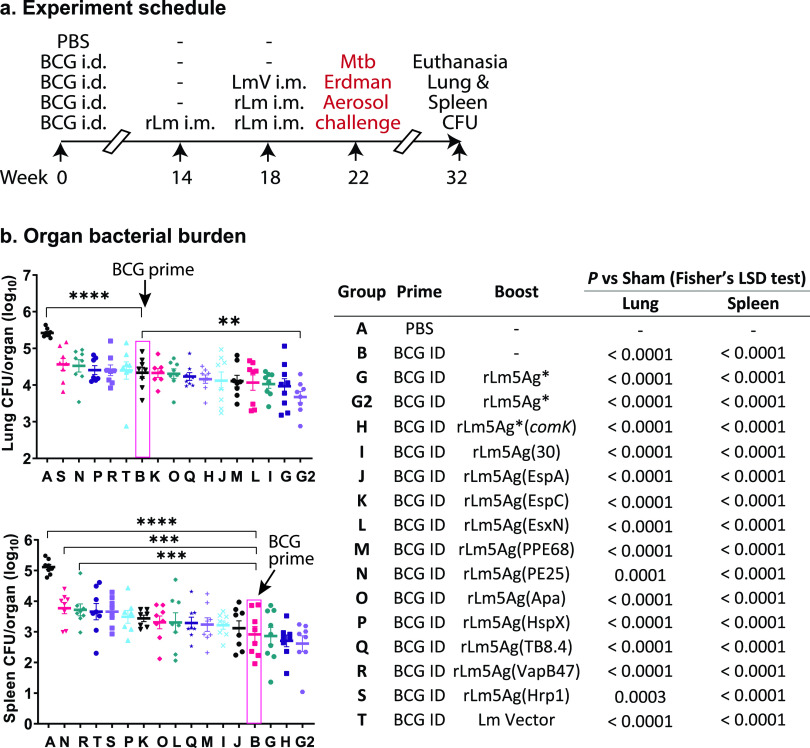
Efficacy against M. tuberculosis aerosol challenge of priming mice with BCG and boosting them with recombinant attenuated L. monocytogenes expressing 5 M. tuberculosis antigens. (a) Experimental schedule. BALB/c mice (8/group) were immunized i.d. with PBS (sham) or 5 × 10^5^ CFU of BCG at week 0. Mice immunized i.d. with BCG were either not boosted or boosted once at week 18 or twice at weeks 14 and 18 (group G2 only) with 2 × 10^6^ CFU of rLm5Ag vaccine candidates. At week 22, the mice were challenged with aerosolized M. tuberculosis Erdman strain (average of 19 CFU delivered to the lungs of each animal, as assayed at day 1 postchallenge to mice), and at week 32, 10 weeks postchallenge, mice were euthanized. (b) Organ bacterial burden. Lungs (top) and spleens (bottom) of mice in the vaccinated and challenge groups (described in the table to the right of the graphs) were removed and assayed for bacillus burdens. Each symbol represents one mouse, and the means ± SEM are shown as bars. Group designations are listed beneath the horizontal axis. The pink-colored boxes indicate group B, BCG-primed only. The log_10_ CFU in the lungs and spleens were compared to group A (sham) and group B (BCG i.d.) by one-way ANOVA with Fisher’s LSD test. The significant *P* values to group B (BCG i.d.) in the lungs and spleens are shown in the graph; **, *P < *0.01; ***, *P < *0.001; and ****, *P < *0.0001. The significant *P* values between sham group (A) and all other groups are listed in the table to the right.

To screen for optimal M. tuberculosis antigens, we examined the protective efficacy against aerosolized M. tuberculosis by priming mice i.d. with BCG and boosting them i.m. (this experiment was initiated prior to our obtaining results of the experiment described above that determined that the optimal route was s.c.) with the 11 new rLm5Ag vaccine candidates, each expressing the M. tuberculosis 4Ag fusion protein (Mpt64-TB10.4-ESAT6-CFP10) ligated at its C terminus with a new 5th antigen. We immunized BALB/c mice, 8 per group, i.d. with PBS (sham) or i.d. with 5 × 10^5^ CFU of BCG at week 0 and boosted them i.m. once at week 18 with 2 × 10^6^ CFU each of the L. monocytogenes vector, 11 rLm5Ag candidates, or with 1 of 2 recombinant attenuated L. monocytogenes vaccine combinations expressing the same 5 M. tuberculosis antigens as rLm5Ag(30). At week 22, we challenged the mice with aerosolized M. tuberculosis (average of 19 CFU delivered to the lungs of each animal). At week 32 (10 weeks postchallenge), we euthanized the mice and assayed bacillus burdens in their lungs and spleens (Fig. 4a). Among the groups tested (Fig. 4b, right), group G (rLm4Ag with rLm30) served as a control for group I [rLm5Ag(30)] for comparison of the combination of two recombinant attenuated L. monocytogenes vaccines (rLm4Ag plus rLm30) with a single vaccine, rLm5Ag(30), expressing M. tuberculosis 4Ag fused with r30; group G2 served as a control for group G for comparison of 1 (week 18) versus 2 (week 14 and week 18) boosts with this same combination of recombinant attenuated L. monocytogenes vaccines; group H served as a control for group G for comparison of M. tuberculosis antigens expressed from the *comK* locus [rLm4Ag(*comK*) plus rLm30(*comK*)] versus the *tRNA^arg^* locus (rLm4Ag plus rLm30) in the Lm chromosome. As shown in Fig. 4b, BCG immunization alone gave a high level of protection, 1.1-log_10_ CFU in the lung (*P < *0.0001) and 2.2-log_10_ CFU in the spleen (*P < *0.0001) versus sham-immunized mice. Also as shown in Fig. 4b, (i) the L. monocytogenes vector (group T) does not boost protective immunity induced by BCG alone; (ii) among the 13 recombinant attenuated L. monocytogenes vaccines screened, the recombinant attenuated L. monocytogenes vaccine candidates expressing 4Ag plus r30 in various forms, i.e., rLm4Ag plus rLm30, rLm4Ag(*comK*) plus rLm30(*comK*), and rLm5Ag(30) in groups G, G2, H, and I, are the best booster vaccines; the lung CFU in the mice boosted with these vaccines are lower than those in mice primed i.d. with BCG only, although the difference did not reach statistical significance for a single booster immunization (however, boosting twice with rLm4Ag plus rLm30 induced immunoprotection significantly more greatly than BCG alone in the lung [*P* < 0.01]); (iii) immunity induced by M. tuberculosis 5Ag expressed from a single recombinant attenuated L. monocytogenes vaccine [rLm5Ag(30); group I] is comparable to that induced by the same M. tuberculosis 5Ags expressed by two recombinant attenuated L. monocytogenes vaccines administered together (rLm4Ag plus rLm30; group G), as evidenced by the equivalent CFU in the lungs of mice in these two groups; (iv) immunity induced by the combination of two vaccines (rLm4Ag plus rLm30) expressing M. tuberculosis 5Ag from the *comK* locus (group H) is comparable to that induced by the combination of two parallel vaccines expressing M. tuberculosis 5Ag from the *tRNA^arg^* locus (group G); and (v) as noted, two boosts with rLm5Ag (rLm4Ag plus rLm30) (group G2) are more efficacious than one boost (group G), as was similarly demonstrated in the experiment shown in Fig. 2a. Of note, boosting BCG twice with rLm5Ag* reduced CFU in the lung by 1.75-log_10_ CFU (*P < *0.0001) and in the spleen by 2.2-log_10_ CFU (*P < *0.0001) compared with sham-immunized mice.

Overall, of the single vaccines expressing 5 M. tuberculosis antigens, we considered rLm5Ag(30) as the most efficacious, as it had the lowest CFU count in the lung, the major site of TB pathology, and the second-lowest CFU count in the spleen. Moreover, the 5 antigens expressed by the vaccine were shown to significantly enhance the level of protective immunity conferred by BCG alone in three independent experiments. Hence, this vaccine was evaluated further for immunogenicity.

### Boosting BCG-primed mice with rLm5Ag(30) induces disparate antigen-specific CD4^+^ and CD8^+^ T-cell responses and serum antibody responses in C57BL/6 and BALB/c mice.

To determine the immunogenicity of rLm5Ag(30) as a booster vaccine in BCG-primed mice, we primed C57BL/6 and BALB/c mice, 4/group, i.d. with 5 × 10^5^ CFU of BCG at week 0 and boosted them s.c. twice at weeks 14 and 18 with L. monocytogenes vector or rLm5Ag(30). Six days after the last immunization, we anesthetized the mice, bled and euthanized them, prepared single-cell suspensions of spleen and lung cells, seeded the cells in 96-well cell culture plates, stimulated the cells with various M. tuberculosis antigens, and assayed T-cell immunity by intracellular cytokine staining (ICS). Immune sera were assayed for serum IgG to M. tuberculosis proteins and formalin-killed rLm5Ag(30) (FK-rLm5Ag).

**FIG 5:**
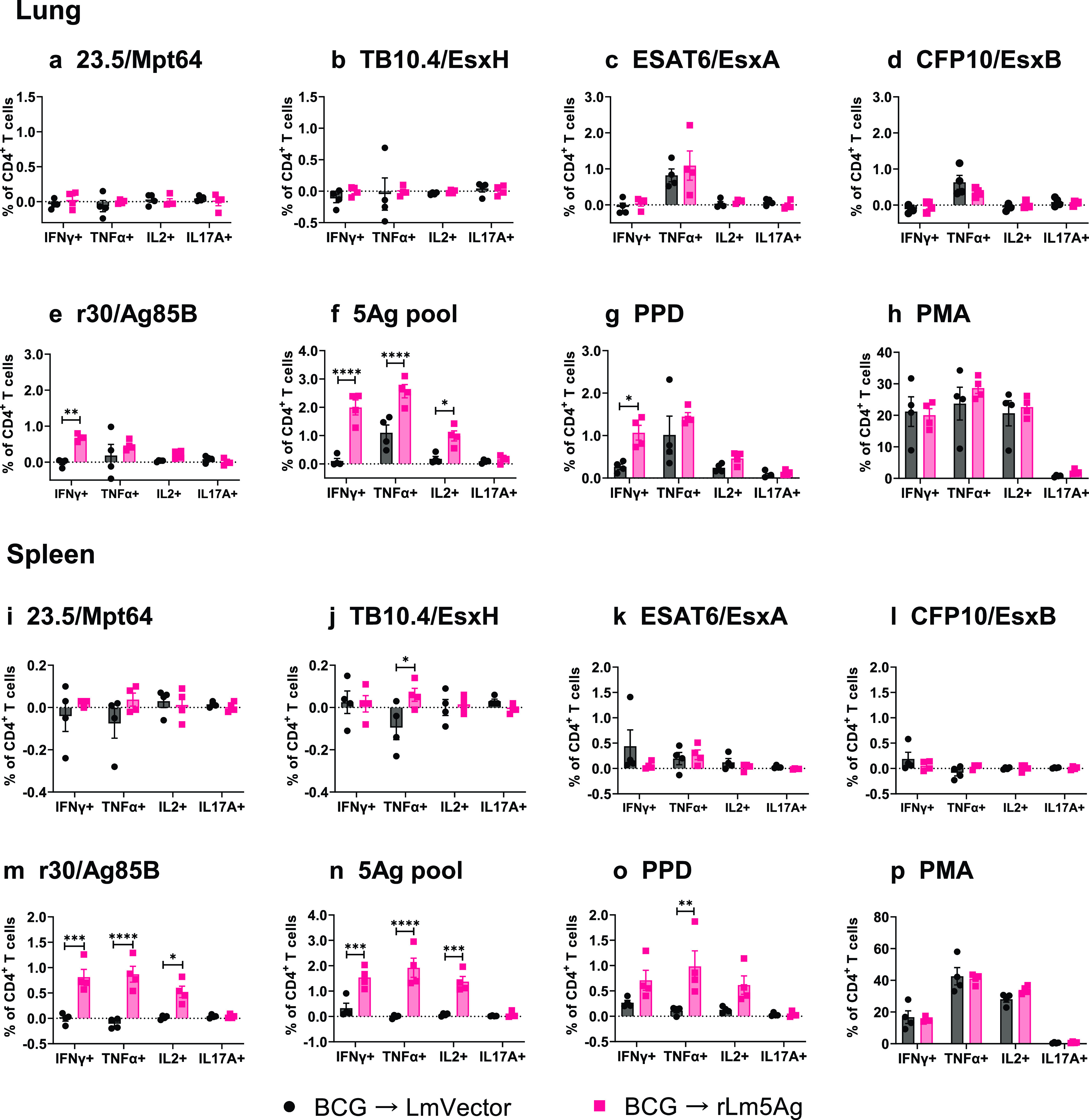
Frequency of cytokine-expressing CD4^+^ T cells in the lungs and spleens of C57BL/6 primed with BCG and boosted twice with L. monocytogenes vector or rLm5Ag(30). Mice (*n* = 4/group) were primed with BCG at week 0 and boosted twice at weeks 14 and 18 with L. monocytogenes vector (black bars and symbols) or rLm5Ag(30) (rLm5Ag) (pink bars and symbols) expressing Mpt64-TB10.4-ESAT6-CFP10-r30. Six days after the last immunization, mice were euthanized, their lungs and spleens removed, and single-cell suspensions prepared and stimulated with recombinant proteins 23.5/Mpt64 (a, i), TB10.4/EsxH (b, j), ESAT6/EsxA (c, k), CFP10/EsxB (d, l), r30/Ag85B (e, m), pool of the 5Ags (f, n), PPD (g, o), or PMA (positive control) (h, p) in the presence of anti-CD28 monoclonal antibody for 6 h (except in the case of PMA [positive control] for 4 h), and the cells were assayed by intracellular cytokine staining (ICS) for surface markers of CD4 and intracellular markers of IFN-γ, TNF-α, IL-2, and IL-17A, as indicated below each panel. Each symbol represents one animal. Values are the mean ± SEM. *, *P < *0.05; **, *P < *0.01; ***, *P < *0.001; ****, *P < *0.0001 by two-way ANOVA with Sidak’s post-multiple-comparison test.

**FIG 6:**
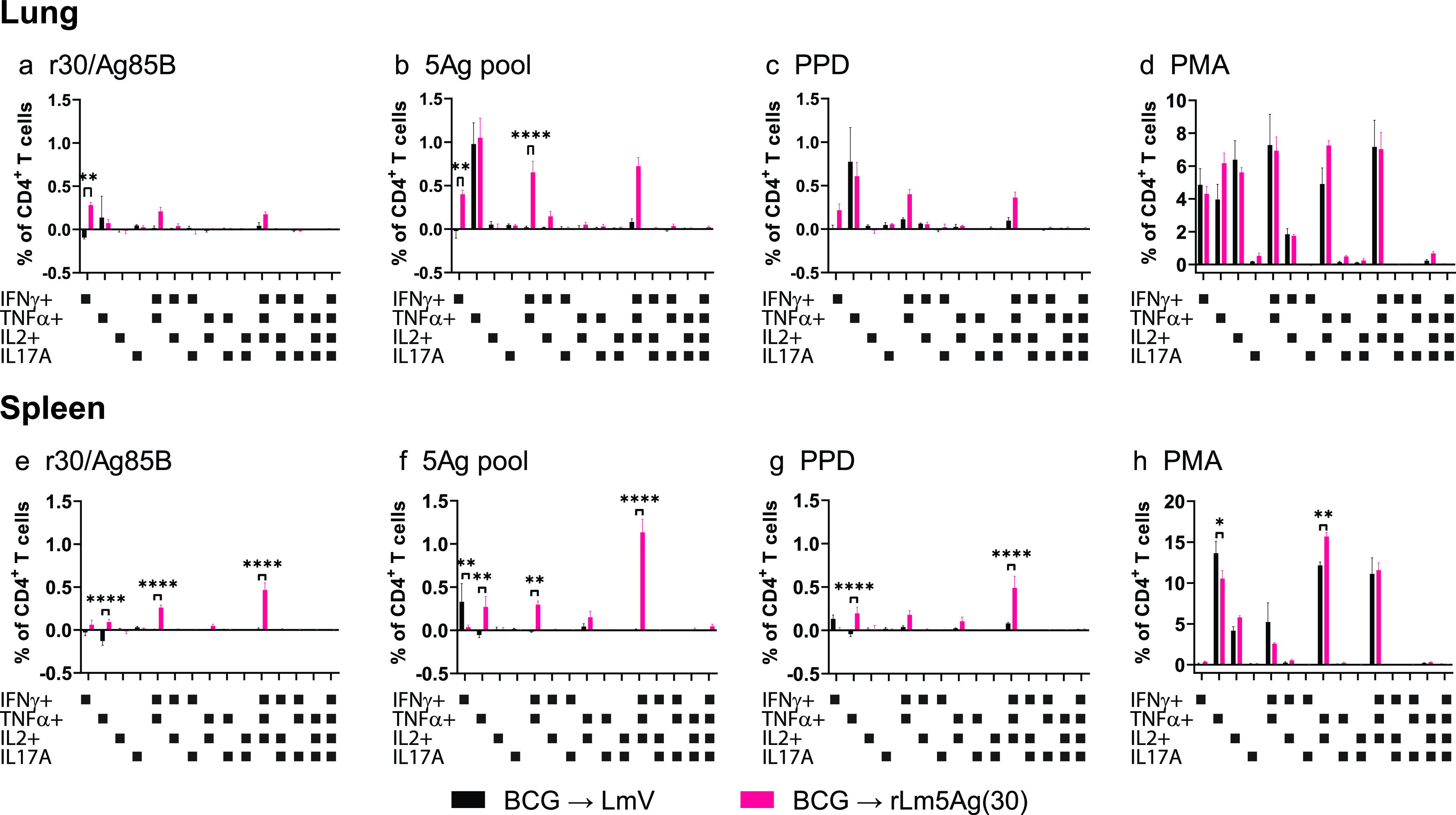
Frequency of polyfunctional cytokine-expressing CD4^+^ T cells in the lungs and spleens of C57BL/6 mice primed with BCG and boosted twice with L. monocytogenes vector or rLm5Ag(30). Mice were immunized, and lung and spleen cells were prepared and stimulated with recombinant proteins as described in the legend to Fig. 5. The cells were assayed by ICS for surface markers of CD4 and intracellular markers of IFN-γ, TNF-α, IL-2, and IL-17A. The frequencies of CD4^+^ T cells expressing 1 or combinations of 2, 3, or 4 of the four cytokines assayed in response to stimulation with r30/Ag85B (a, e), pool of the 5Ags (b, f), PPD (c, g), or PMA (positive control) (d, h) are shown, as indicated below each panel. Values are the mean ± SEM. *, *P < *0.05; **, *P < *0.01; ****, *P < *0.0001 by two-way ANOVA with Sidak’s post-multiple-comparison test.

C57BL/6 mice, but not BALB/c mice, primed-boosted with BCG and rLm5Ag(30), produced a lower frequency of CD4^+^ T cells, greater frequency of CD8^+^ T cells, and lower CD4^+^/CD8^+^ T cell ratio than mice primed-boosted with BCG-L. monocytogenes vector in their lungs after *in vitro* stimulation without (medium control) or with M. tuberculosis antigens (Fig. S5). There are no significant differences in the frequencies of CD4^+^ and CD8^+^ T cells in the spleens of C57BL/6 and BALB/c mice (Fig. S6). With respect to CD4^+^ T cells in the lungs and spleens of C57BL/6 mice, as shown in Fig. 5 and 6, mice primed with BCG and boosted with rLm5Ag(30) produce significantly greater frequencies of CD4^+^ T cells expressing intracellular cytokines IFN-γ, TNF-α, and/or IL-2 (Fig. 5) and polyfunctional CD4^+^ T cells expressing IFN-γ and TNF-α, or IFN-γ, TNF-α, and IL-2 (Fig. 6) in response to *in vitro* stimulation with r30/Ag85B, 5Ag pool, purified protein derivative (PPD), or TB10.4/EsxH than mice immunized with the L. monocytogenes vector. No significant differences in the frequencies of CD4^+^ T cells expressing any of the cytokines were detected after *in vitro* stimulation with ESAT6/EsxA, CFP10/EsxB, and 23.5/Mpt64. As expected, mice immunized with the L. monocytogenes vector and rLm5Ag(30) produced mostly comparable amounts of cytokines after *in vitro* stimulation with PMA (Fig. 5 and 6).

10.1128/mbio.00687-22.5 FIG S5 Frequency of CD4^+^ and CD8^+^ T cells in the lungs of C57BL/6 and BALB/c mice primed with BCG and boosted twice with the L. monocytogenes vector or rLm5Ag(30). C57BL/6 (left) and BALB/c (right) mice (*n* = 4/group) were primed with BCG at week 0 and boosted twice at weeks 14 and 18 with L. monocytogenes vector (black bars) or rLm5Ag(30) (pinks bars) expressing the 5Ag fusion protein Mpt64-EsxH-EsxA-EsxB-r30. Six days after the last immunization, mice were euthanized, their lungs and spleens were removed, single-cell suspensions were prepared and stimulated with recombinant proteins (as indicated beneath the *x* axis) in the presence of anti-CD28 monoclonal antibody for 6 h (except in the case of PMA [positive control] for 4 h), and the cells were assayed by exclusion of dead cells and staining for surface markers of CD4 and CD8 T cells. Frequencies of CD4^+^ (top) and CD8^+^ T cells (middle) and the ratio of CD4^+^ to CD8^+^ T cells (bottom) are shown. Values are the means ± SEM. *, *P < *0.05; **, *P < *0.01; ***, *P < *0.01; ****, *P < *0.0001 by two-way ANOVA with Sidak’s post-multiple-comparison test (Prism v9.02). Download FIG S5, EPS file, 2.5 MB. Copyright © 2022 Jia et al. 2022 Jia et al. https://creativecommons.org/licenses/by/4.0/ This content is distributed under the terms of the Creative Commons Attribution 4.0 International license.

10.1128/mbio.00687-22.6 FIG S6 Frequency of CD4^+^ and CD8^+^ T cells in the spleens of C57BL/6 and BALB/c mice primed with BCG and boosted twice with the L. monocytogenes vector or rLm5Ag(30). C57BL/6 (left) and BALB/c (right) and mice (*n* = 4/group) were primed-boosted with BCG and L. monocytogenes vector (black bars) or BCG and rLm5Ag30 (pink bars), their lungs and spleens were removed, single-cell suspensions were stimulated with protein antigens (as indicated beneath the *x* axis), and cells were analyzed by ICS as described in the legend to Fig. S5. Frequencies of CD4^+^ (top) and CD8^+^ T cells (middle) and the ratio of CD4^+^ to CD8^+^ T cells (bottom) are shown. Values are the means ± SEM. Download FIG S6, EPS file, 2.4 MB. Copyright © 2022 Jia et al. 2022 Jia et al. https://creativecommons.org/licenses/by/4.0/ This content is distributed under the terms of the Creative Commons Attribution 4.0 International license.

**FIG 7:**
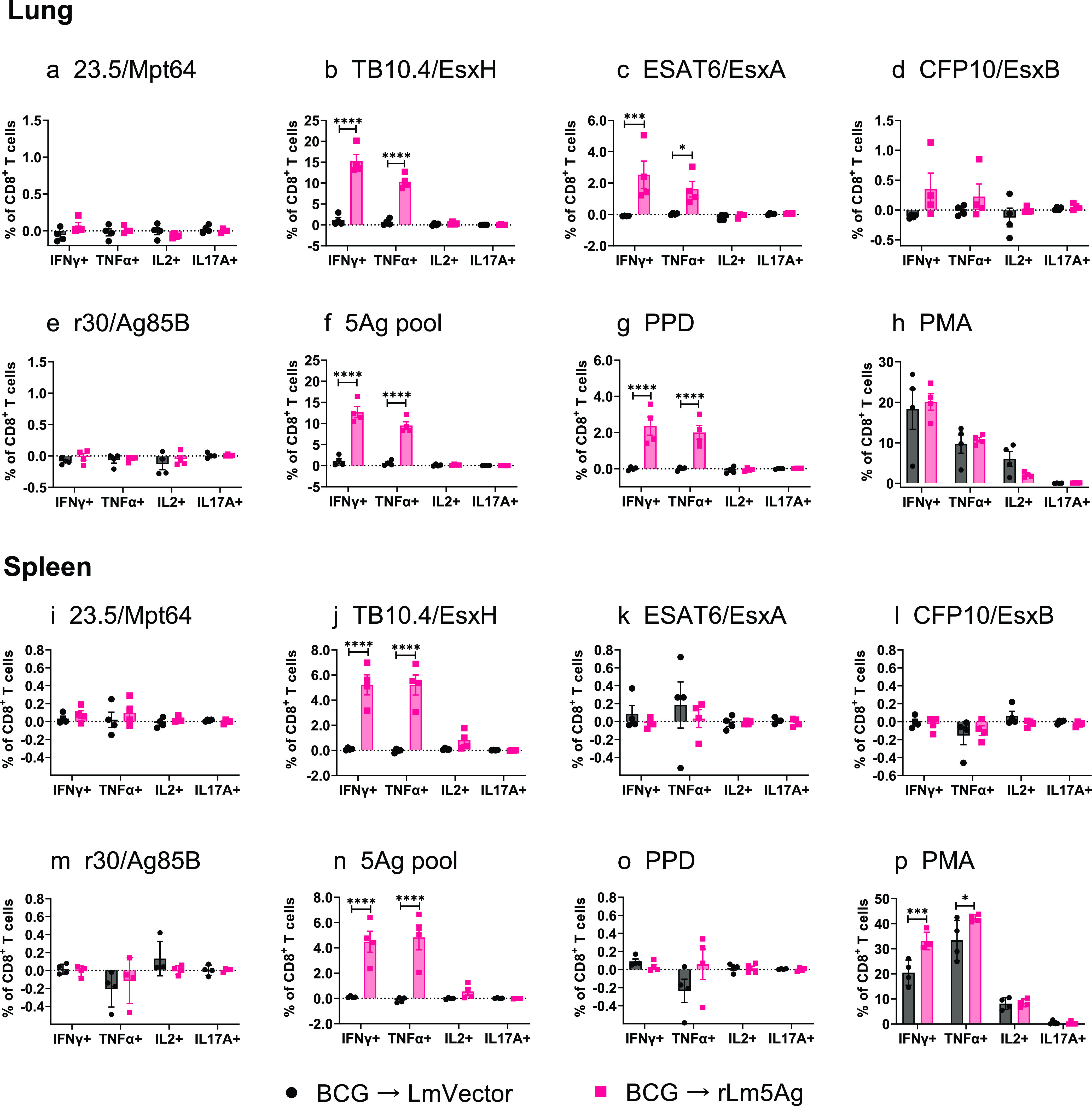
Frequency of cytokine-expressing CD8^+^ T cells in the lungs and spleens of C57BL/6 mice primed with BCG and boosted twice with L. monocytogenes vector or rLm5Ag(30). Mice were immunized, and lung and spleen cells were prepared and stimulated with recombinant proteins as described in the legend to Fig. 5. The cells were assayed by ICS for surface markers of CD8 and intracellular markers of IFN-γ, TNF-α, IL-2, and IL-17A. The frequencies of CD8^+^ T cells expressing IFN-γ, TNF-α, IL-2, and IL-17A in response to stimulation with recombinant proteins 23.5/Mpt64 (a, i), TB10.4/EsxH (b, j), ESAT6/EsxA (c, k), CFP10/EsxB (d, l), r30/Ag85B (e, m), pool of the 5Ags (f, n), PPD (g, o), and PMA (positive control) (h, p) are shown. Each symbol represents one animal. Black bars and symbols, primed-boosted with BCG and L. monocytogenes vector; pink bars and symbols, primed-boosted with BCG and rLm5Ag(30). Values are the mean ± SEM. *, *P < *0.05; ***, *P < *0.001; ****, *P < *0.0001 by two-way ANOVA with Sidak’s post-multiple-comparison test.

With respect to CD8^+^ T cells in lungs and spleens of C57BL/6 mice (Fig. 7), mice primed-boosted with BCG-rLm5Ag(30) produced significantly greater frequencies of lung and spleen CD8^+^ T cells expressing IFN-γ and TNF-α in response to *in vitro* stimulation with the TB10.4/EsxH (*P* < 0.0001 for both cytokines in both organs) (Fig. 7b and j) and the 5Ag pool (*P* < 0.0001 for both cytokines in both organs) (Fig. 7f and n) than mice immunized with the L. monocytogenes vector. Two notable differences between lungs and spleens were that mice primed-boosted with BCG-rLm5Ag(30) produced significantly greater frequencies of CD8^+^ T cells expressing IFN-γ and TNF-α in response to *in vitro* stimulation with the ESAT6/EsxA (*P < *0.05 and *P < *0.001, respectively) (Fig. 7c) and PPD (*P* < 0.0001) (Fig. 7g) for cells from their lungs but not their spleens (Fig. 7k and o), while mice primed-boosted with BCG-rLm5Ag(30) produced significantly greater frequencies of CD8^+^ T cells expressing IFN-γ and TNF-α for cells from their spleens (*P* < 0.001 and *P < *0.05, respectively) (Fig. 7p), but not their lungs (Fig. 7h), in response to *in vitro* stimulation with PMA than mice immunized with the L. monocytogenes vector; the relative differences in frequency and in statistical significance were much less than for 5Ag and TB10.4/EsxH (Fig. 7j and n). No significant differences in the frequencies of lung and spleen CD8^+^ T cells expressing any of the cytokines were detected after *in vitro* stimulation with 23.5/Mpt64 (Fig. 7a and i), r30/Ag85B (Fig. 7e and m), or CFP10/EsxB (Fig. 7d and l). These results show that boosting BCG-primed C57BL/6 mice with rLm5Ag(30) induces M. tuberculosis antigen-specific CD4^+^ and CD8^+^ T cell-mediated immune responses.

**FIG 8:**
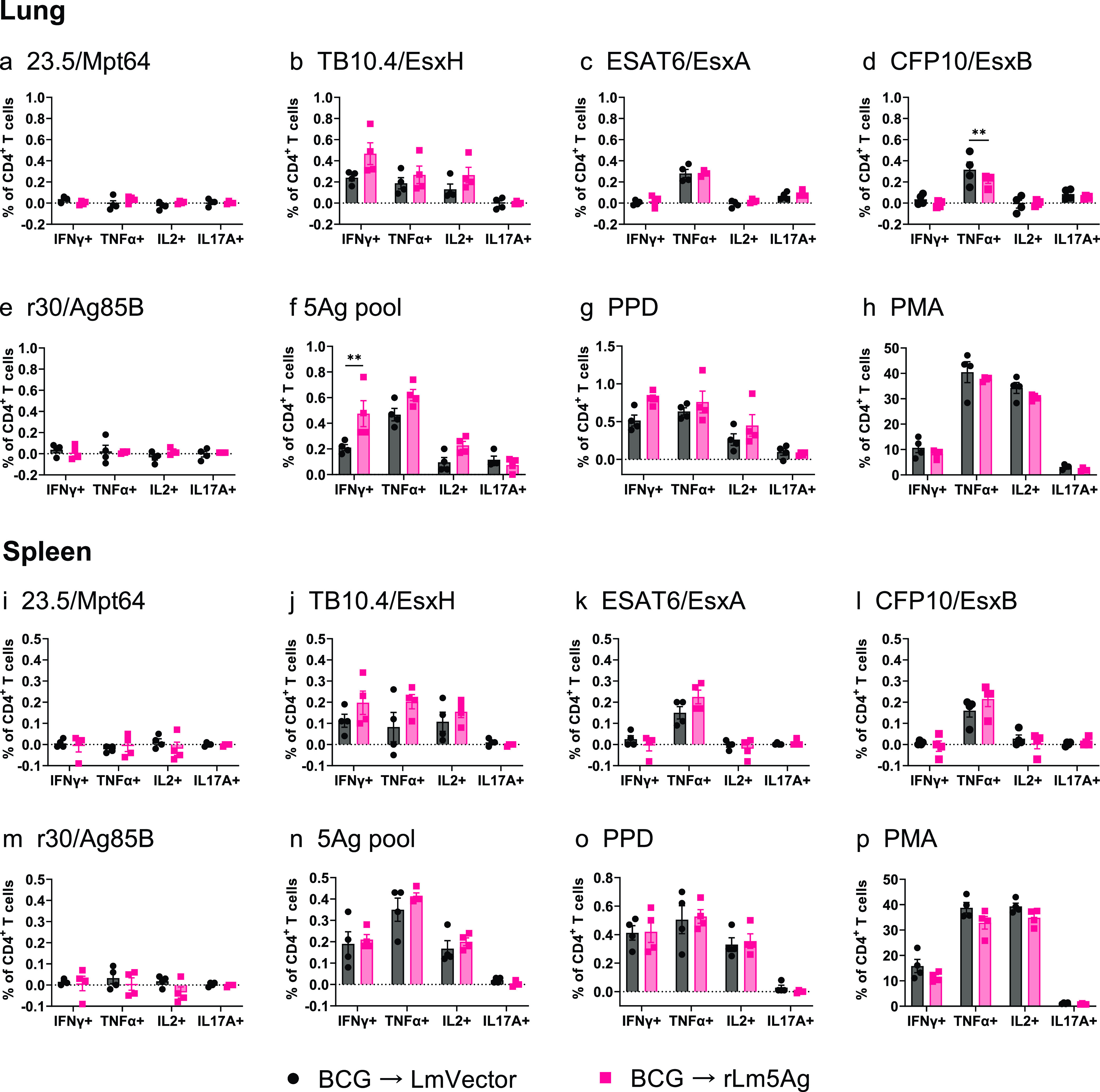
Frequency of cytokine-expressing CD4^+^ T cells in the lungs and spleens of BALB/c mice primed with BCG and boosted twice with L. monocytogenes vector or rLm5Ag(30). BALB/c mice (*n* = 4/group) were primed with BCG at week 0 and boosted twice at weeks 14 and 18 with L. monocytogenes vector (black bars and symbols) or rLm5Ag(30) (rLm5Ag) (pink bars and symbols) expressing 23.5-TB10.4-ESAT6-CFP10-r30. Six days after the last immunization, mice were euthanized, their spleens and lungs were removed, and single-cell suspensions were prepared and stimulated with recombinant proteins 23.5/Mpt64 (a, i), TB10.4/EsxH (b, j), ESAT6/EsxA (c, k), and CFP10/EsxB (d, l), r30/Ag85B (e, m), pool of the 5Ags (f, n), PPD (g, o), or PMA (positive control) (h, p) and in the presence of anti-CD28 monoclonal antibody for 6 h (except in the case of PMA [positive control] for 4 h), and the cells were assayed by intracellular cytokine staining (ICS) for surface markers of CD4 and intracellular markers of IFN-γ, TNF-α, IL-2, and IL-17A, as indicated below each panel. Each symbol represents one animal. Values are the mean ± SEM. **, *P < *0.01 by two-way ANOVA with Sidak’s post-multiple-comparison test.

**FIG 9:**
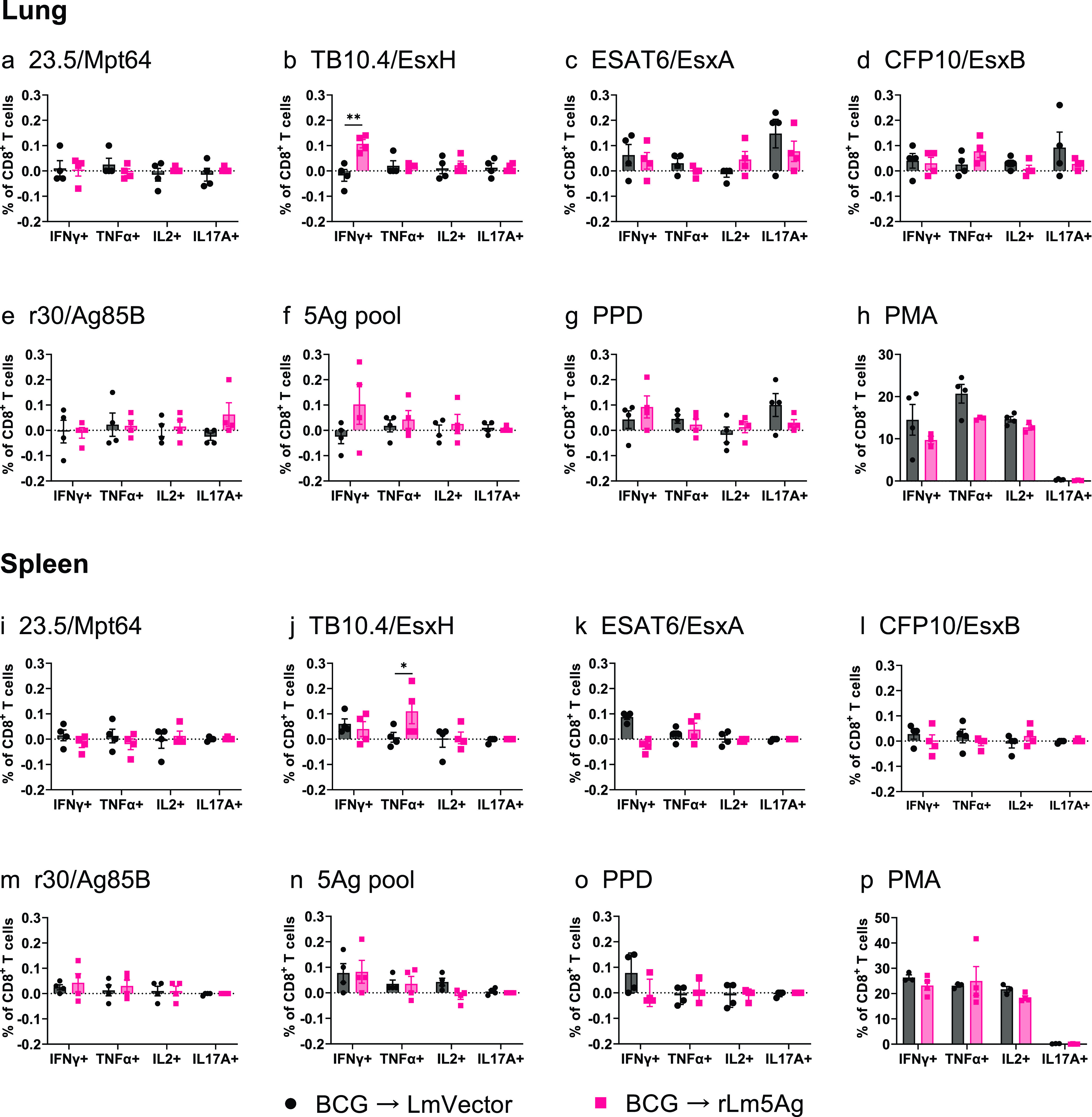
Frequency of cytokine-expressing CD8^+^ T cells in the lungs and spleens of BALB/c mice primed with BCG and boosted twice with L. monocytogenes vector or rLm5Ag(30). BALB/c mice were immunized, and spleen and lung cells were prepared and stimulated with recombinant proteins as described in the legend to Fig. 8. The cells were assayed by ICS for surface markers of CD8 and intracellular markers of IFN-γ, TNF-α, IL-2, and IL-17A. The frequencies of CD8^+^ T cells expressing IFN-γ, TNF-α, IL-2, and IL-17A in response to stimulation with recombinant proteins 23.5/Mpt64 (a, i), TB10.4/EsxH (b, j), ESAT6/EsxA (c, k), and CFP10/EsxB (d, l), r30/Ag85B (e, m), pool of the 5Ags (f, n), PPD (g, o), or PMA (positive control) (h, p) are shown. Each symbol represents one animal. Black bars and symbols, primed-boosted with BCG and L. monocytogenes vector; pink bars and symbols, primed-boosted with BCG and rLm5Ag(30). Values are the mean ± SEM. *, *P < *0.05; **, *P < *0.01 by two-way ANOVA with Sidak’s post-multiple-comparison test.

In a similar experiment performed in BALB/c mice (Fig. 8), in contrast to C57BL/6 mice, there were no significant differences between mice primed-boosted with BCG-L. monocytogenes vector and BCG-rLm5Ag(30) in frequencies of lung and splenic CD4^+^ and CD8^+^ T cells expressing IFN-γ, TNF-α, IL-2, or IL-17 in response to *in vitro* stimulation with 23.5, TB10.4, ESAT6, r30/Ag85B, PPD, and PMA; the only exceptions were that mice primed-boosted with BCG-rLm5Ag(30) had significantly greater frequencies of lung CD4^+^ T cells expressing IFN-γ in response to stimulation with the 5Ag pool (*P < *0.01) (Fig. 8f), and mice primed-boosted with BCG-rLm5Ag(30) had significantly greater frequencies of lung CD4^+^ T cells expressing TNF-α in response to stimulation with CFP10 (*P* < 0.01) (Fig. 8d). With respect to CD8^+^ T cells (Fig. 9), compared with mice primed-boosted with BCG-L. monocytogenes vector, BALB/c mice primed-boosted with BCG-rLm5Ag(30) produced significantly greater frequencies of lung and spleen CD8^+^ T cells expressing IFN-γ or TNF-α in response to TB10.4 (*P < *0.01 for lung cells [Fig. 9b] and *P < *0.05 for spleen cells [Fig. 9j]), but not to other M. tuberculosis antigens expressed by the rLm5Ag(30) vaccine (Fig. 9). These results indicate the rLm5Ag(30) multiantigenic vaccine candidate induces a qualitatively and quantitatively different immune response in BCG-primed C57BL/6 and BALB/c mice.

With respect to the humoral immune response induced by BCG-rLm5Ag primed-boost vaccination, as shown in Fig. S7, C57BL/6 mice primed-boosted with BCG-rLm5Ag(30) produced serum IgG antibodies specific to FK-rLm5Ag(30), but not to individual M. tuberculosis protein antigens (Ag85B, EsxA, EsxH, Mpt64, and PPD), at a level significantly higher than mice primed-boosted with BCG-L. monocytogenes vector (Fig. S7a). There were no significant differences in serum antibody titers to any of the antigens between BALB/c mice primed-boosted with BCG-rLm5Ag(30) or BCG-L. monocytogenes vector (Fig. S7b). These results indicate that boosting BCG-immunized mice with rLm5Ag(30) has a negligible or very limited impact on the humoral immune response to M. tuberculosis antigens.

10.1128/mbio.00687-22.7 FIG S7 Serum IgG antibody in C57BL/6 and BALB/c mice primed with BCG and boosted twice with the L. monocytogenes vector or rLm5Ag(30). C57BL/6 (top) and BALB/c (bottom) mice (*n* = 4/group) were primed-boosted with BCG and L. monocytogenes vector (LmV) (black bars) or BCG and rLm5Ag(30) (rLm5Ag) (pink bars), anesthetized, bled, and euthanized; their sera were isolated, incubated with M. tuberculosis protein antigens or with formalin-killed rLm5Ag(30) (FK-rLm5Ag) as indicated beneath the *x* axis, and assayed for endpoint titer of antigen-specific IgG antibody. Values are the means ± SEM. *, *P < *0.05 by two-way ANOVA with Sidak’s post-multiple-comparison test (Prism v9.2.0). Download FIG S7, EPS file, 1.7 MB. Copyright © 2022 Jia et al. 2022 Jia et al. https://creativecommons.org/licenses/by/4.0/ This content is distributed under the terms of the Creative Commons Attribution 4.0 International license.

## DISCUSSION

Our study shows that boosting BCG-primed C57BL/6 and BALB/c mice with an Lm-vectored multiantigenic M. tuberculosis vaccine candidate expressing combinations of M. tuberculosis proteins, especially rLm5Ag(30), expressing a fusion protein of r30/Ag85B, TB10.4/EsxH, ESAT6/EsxA, CFP10/EsxB, and 23.5/Mpt64, enhances the immunoprotection conferred by BCG against aerosol challenge with the virulent M. tuberculosis Erdman strain in both mouse strains. Boosting C57BL/6 mice with rLm5Ag(30) significantly enhances the level of CD8^+^ T cell expression in the lungs and spleen, the frequency of polyfunctional splenic CD4^+^ T cells expressing IFN-γ, TNF-α, and IL-2 in response to r30/Ag85B, PPD, and the 5Ag pool, and the frequency of lung and spleen CD8^+^ T cells expressing IFN-γ and TNF-α in response to TB10.4/EsxH, ESAT6/EsxA, the 5Ag pool, and PPD. Although boosting BCG-primed BALB/c mice with rLm5Ag(30) also enhances the frequency of some cytokine-secreting lymphocytes, specifically lung CD4^+^ T cells expressing IFN-γ or TNF-α in response to the 5 Ag pool or CFP10 and splenic CD8^+^ T cells expressing IFN-γ or TNF-α in response to TB10.4/EsxH antigen, the response is much more limited than in BCG-primed C57BL/6 mice.

Of the five recombinant M. tuberculosis antigens expressed by rLm5Ag(30), all have previously been demonstrated to be immunoprotective individually as well as in combination with other M. tuberculosis antigens. r30/Ag85B has been demonstrated to be highly protective when administered as an adjuvanted recombinant protein (9) or when expressed by recombinant BCG (rBCG30) (4, 41) or an L. monocytogenes vector (22) in guinea pigs and mice. TB10.4 alone or as part of an Ag85B-TB10.4 fusion protein in adjuvant has been shown to induce protection in mice (31) and guinea pigs (42, 43). ESAT6, alone or in combination with antigen 85B, administered with the adjuvant monophosphoryl lipid A, has been shown to induce protective immunity in mice (29, 32). CFP10 delivered via a DNA vaccine induces protection against aerosolized M. tuberculosis Erdman in C3H/HeJ mice (30), and a Salmonella-vectored vaccine expressing an ESAT6-CFP10 fusion protein protects C57BL/6 mice against aerosolized M. tuberculosis H37Rv (44). Finally, the 23.5/Mpt64 protein expressed by a DNA vaccine (25) or surface expressed by recombinant BCG (26) has been found to induce protective immunity in C57BL/6 mice challenged intravenously with H37Rv (25) or by aerosol with M. tuberculosis Erdman (26).

Notably, of the five antigens in rLm5Ag(30), three are absent from BCG entirely (ESAT6/EsxA and CFP10/EsxB) or from modern strains of BCG (23.5/Mpt64). Hence, boosting BCG with rLm5Ag(30) not only potentially enhances the level of immunity to immunoprotective proteins present in BCG but additionally broadens the potential immune response to encompass antigens present in M. tuberculosis but absent from BCG.

Also of note, the five proteins comprising rLm5Ag(30) are all secreted or extracellularly released proteins. Such extracellular proteins have been demonstrated to be especially important immunoprotective antigens of intracellular pathogens and hypothesized early on to play a central role in vaccines against such pathogens, including Legionella pneumophila and Mycobacterium tuberculosis (45, 46).

Our screen of 11 *Listeria*-vectored vaccines expressing 5 M. tuberculosis antigens, all comprising a fusion protein of Mpt64-TB10.4-ESAT6-CFP10 plus 1 of 11 additional antigens, revealed several vaccine candidates that induced protection better than BCG and almost comparable to rLm5Ag(30). The most potent alternative “fifth” antigens were EsxN, PPE68, EspA, and TB8.4. Of these, one antigen, PPE68, is absent from BCG. We have subsequently constructed a 9-antigen *Listeria*-vectored vaccine incorporating these additional four M. tuberculosis antigens, and in ongoing studies, we are evaluating it for protective efficacy in mice, guinea pigs, and nonhuman primates.

The rLm5Ag(30) vaccine induced significantly enhanced levels of antigen-specific cytokine-secreting CD4^+^ and CD8^+^ T cells in BCG-immunized C57BL/6, but not in BCG-immunized BALB/c, mice, where such responses were weak and sporadic, reflecting the well-established Th1 bias of C57BL/6 mice versus the Th2 bias of BALB/c mice. Similarly, BCG immunization alone has been found to induce a greater Th1-type response in C57BL/6 than BALB/c mice (47, 48), although this has not been observed universally (49). In any case, despite the disparate immune responses induced by the rLm5Ag(30) vaccine in BCG-immunized C57BL/6 and BALB/c mice, recombinant attenuated L. monocytogenes vaccines expressing these five antigens boosted protection against M. tuberculosis aerosol challenge in both mouse strains. This result mirrors previous observations that differences in the ability of these two mouse strains to generate Th1 helper cells are not reflected by differences in their ability to resist M. tuberculosis infection (50, 51). In an interesting twist on the subject, Garcia-Pelayo et al. found that in mice immunized i.d. with BCG Danish, BALB/c mice developed a stronger TH1 response than C57BL/6 mice in both lung and spleen cells after stimulation with a cocktail of M. tuberculosis-secreted proteins; nevertheless, both mouse strains showed equivalent protection against virulent Mycobacterium bovis challenge (49). Clearly, TH1 type responses are important for immunoprotection against M. tuberculosis, as evidenced by the increased susceptibility to M. tuberculosis challenge of IFN-γ knockout mice and the enhancement in protection against M. tuberculosis challenge in BALB/c mice administered IL-12 (52, 53). However, taken together, the aforementioned studies showing comparable resistance of BALB/c and C57BL/6 mice to virulent mycobacterial challenge, albeit conducted under different experimental conditions, indicate that additional immune mechanisms, aside from the strength of the TH1 response, are also important.

Of note, in all three experiments in which they were evaluated, recombinant attenuated L. monocytogenes vaccines expressing the 5 M. tuberculosis antigens in rLm5Ag(30) significantly boosted the level of protection conferred by BCG despite the already high level of protection conferred by BCG alone (1.1- to 1.3-log_10_ CFU in the lungs and 1.8- to 2.2-log_10_ CFU in the spleen). Many TB booster vaccines are able to boost the level of protection afforded by BCG alone only if that level is very small, e.g., <0.3-log_10_ CFU (23). Moreover, in all three of these protection experiments, the reduction in log_10_ CFU versus sham-immunized mice conferred by the BCG prime-recombinant attenuated L. monocytogenes boost was ≥1.75-log_10_ CFU in the lung and/or spleen (1.70- to 1.75-log_10_ CFU in the lung and 2.5- to 2.8-log_10_ CFU in the spleen), a threshold of protection achieved by relatively few TB booster vaccines in BCG-immunized animals (23).

A potential major advantage of a *Listeria*-vectored vaccine, particularly with respect to protein/adjuvant vaccines, is enhanced capacity to induce CD8^+^ T cells. CD8^+^ T cells are required to resist M. tuberculosis infection, as demonstrated by studies in mice employing antibody depletion or TAP1 knockout of CD8^+^ T cells (54–57). Consistent with these observations, adoptive transfer of CD8^+^ T cells enhances resistance to TB (58). Of note, CD8^+^ T cells appear to play a more important role in primates than in rodents (59); hence, efficacy studies in rodents may underestimate the efficacy of *Listeria*-vectored vaccines in nonhuman primates and humans. In current studies, we are evaluating the efficacy of a multiantigenic *Listeria*-vectored vaccine in nonhuman primates.

In conclusion, recombinant attenuated L. monocytogenes multiantigenic vaccines, including the rLm5Ag(30) vaccine, abundantly express M. tuberculosis recombinant proteins in broth and macrophages, stably retain the antigen expression cassettes in the absence of antibiotic selection, and display growth kinetics equivalent to the vector in murine and human macrophages. recombinant attenuated L. monocytogenes vaccines expressing the 5 M. tuberculosis antigens 23.5/Mpt64, TB10.4/EsxH, ESAT6/EsxA, CFP10/EsxB, and r30/antigen 85B consistently and significantly boost the high level of protection conferred by BCG alone in both C57BL/6 and BALB/c mouse models of pulmonary TB. The single rLm5Ag(30) vaccine induces broad CD4^+^ and CD8^+^ T cell responses in the lungs and spleens of mice. Thus, the rLm5Ag(30) vaccine holds substantial promise for future development as a vaccine capable of enhancing the level of protective immunity in the majority of the world’s population, who reside in areas of TB endemicity and are immunized during infancy with BCG.

## MATERIALS AND METHODS

### Ethics statement.

All animals were maintained in a specific-pathogen-free animal facility and used according to protocols approved by the UCLA Institutional Animal Care and Use Committee.

### Cell lines, bacteria, animals, and proteins.

Murine (J774A.1, ATCC TIB-67) and human (THP-1, ATCC TIB-202) monocytes were differentiated into macrophage-like cells and cultured in Dulbecco’s modified Eagle’s medium (DMEM) and RMPI 1640 (RPMI) medium, respectively, containing penicillin (100 μg/mL) and streptomycin (100 U/mL) and supplemented with 10% fetal bovine serum (FBS). Mycobacterium bovis BCG Tice was purchased from Organon. M. tuberculosis Erdman strain (ATCC 35801) was harvested from infected outbred guinea pigs to verify virulence, cultured on 7H11 agar, subjected to gentle sonication to obtain a single-cell suspension, and frozen at −80°C for use in animal challenge experiments. All *Listeria* vector and recombinant *Listeria*-vectored vaccine stocks were grown to mid-log phase in yeast extract broth medium, and the bacteria were collected by centrifugation, resuspended in phosphate-buffered saline (PBS), titrated, and stored in 20% glycerol/PBS at −80°C until use. Six- to 8-week-old female C57BL/6 mice were purchased from Harlan (currently Envigo, Livermore, CA, USA) or Jackson Laboratory (Bar Harbor, ME, USA), and BALB/c mice were purchased from Jackson Laboratory.

The following M. tuberculosis protein reagents were obtained through BEI Resources, NIAID, NIH: Ag85B (gene Rv1886c), purified native protein from strain H37Rv, NR-14857; ESAT-6, recombinant protein reference standard, NR-49424; CFP-10, recombinant protein reference standard, NR-49425; and Mpt64, recombinant protein reference standard, NR-44102. The M. tuberculosis protein TB10.4 (gene Rv0288) was obtained from Aeras (formerly in Rockville, MD, USA).

### Construction and verification of Lm-vectored multiantigenic vaccines.

We constructed *Listeria*-vectored multiantigenic recombinant attenuated L. monocytogenes vaccine candidates using the *Lm* Δ*actA* ΔinlB prfA* vector, as we previously described (22). Briefly, to construct recombinant attenuated L. monocytogenes vaccine candidates expressing M. tuberculosis multiantigenic proteins, we analyzed the protein sequences of the selected 15 M. tuberculosis proteins, removed the predicted signal peptides of TB8.4 (2R-28A) (gene Rv1174c), Apa (2H-39A) (gene Rv1860), r30/Ag85B (2Q-43A) (gene Rv1886c), and 23.5/Mpt64 (1V-23A) (gene Rv1980c) and the internal regions of HspX (121I-128V) (gene Rv2031c), PE25 (66I-73L) (gene Rv2431c), and EspA (111F-193L) (geneRv3616c) that might interfere with protein secretion from the recombinant attenuated L. monocytogenes vaccine constructs; we kept the full-length sequences for TB10.4 (gene Rv0288), EsxN (gene Rv1793), Hrp1 (gene Rv2626c), VapB47 (gene RV3407), EspC (gene 3615c), PPE68 (gene Rv3873), CFP-10 (gene Rv3874), and ESAT-6 (gene Rv3875). We optimized the coding sequence for each of the selected proteins for expression in *Listeria*, purchased them from DNA2.0 (Newark, CA), and assembled the optimized DNAs encoding the indicated multiantigenic proteins with or without a spacer encoding a GGSG or GSSGGSSG flexible linker by traditional molecular cloning methods. We cloned the final assembled DNAs into a phage-based *Listeria* site-specific integration vector derived from pPL1 (kindly provided by P. Lauer) or pPL2e (kindly provided by J. Skoble) (60) downstream of the Lm *actA* promoter and ligated in-frame to the C terminus of the N-terminal 100 amino acids (aa) of ActA (ActAN). Subsequently, we integrated the M. tuberculosis antigen expression cassette vectored by pPL1 and pPL2e into the *comK* and the 3′ end of the *tRNA^arg^* locus, respectively, on the recipient *Listeria* chromosome, as described previously by us (22) and Lauer et al. (17, 60). All molecular plasmid constructs were confirmed by restriction enzyme digestion and nucleotide sequencing. The final M. tuberculosis antigen expression cassette in the recombinant attenuated L. monocytogenes chromosome was verified by PCR using primers NC16 (GTCAAAACATACGCTTCTTATC) and PL95 (ACATAATCAGTCCAAAGTAGATGC) (60), specifically amplifying a unified 548-bp PCR product in strains that contain an integration vector at the bacterial attachment site *tRNA^arg^-attBB′*, and using primers 319 (ACCGACTGGAAACAGGCAAA) and 327 (ACCAAGATACGAAACTGCACG), specifically amplifying a PCR product across the inserted gene with various sizes in different strains; the PCR products were further confirmed by nucleotide sequencing.

### Growth kinetics of recombinant attenuated L. monocytogenes multiantigenic vaccines in broth culture and in murine and human macrophage-like cells.

The growth kinetics of recombinant attenuated L. monocytogenes vaccine candidates in broth culture and macrophage-like cells were examined as described by us previously with modifications (22). Glycerol stocks of the L. monocytogenes vector and recombinant attenuated L. monocytogenes vaccine candidates were inoculated into BHI medium supplemented with streptomycin (200 μg/mL) (the L. monocytogenes vector is streptomycin resistant) to prevent any contamination and grown overnight under stationary conditions in a 37°C incubator with 5% CO_2_. The overnight culture was inoculated into 5 mL fresh BHI with streptomycin at an initial optical density at 540 nm (OD_540_) of ~0.05 and incubated at 37°C with shaking at 180 rpm. At 0, 3, 5, and 7 h postinoculation, a 1-mL aliquot of each culture was removed and measured for OD_540_.

The growth kinetics in macrophage-like cells was assayed by infecting monolayers of murine macrophage-like cells (J774A.1) or phorbol 12-myristate 13-acetate (PMA)-differentiated monolayers of human macrophage-like cells (THP-1) with the L. monocytogenes vector or recombinant attenuated L. monocytogenes candidates cultured overnight to stationary phase at a multiplicity of infection of 1:10 for 90 min in DMEM (J774A.1) or RPMI (THP-1) medium supplemented with 10% heat-inactivated FBS (HI-FBS). After 90 min infection, cells were washed three times with PBS supplemented with 2% HI-FBS. The infected cells were cultured for an additional 4.5 h in DMEM or RPMI medium supplemented with 10% HI-FBS and gentamicin (10 μg/mL). At 0, 2, 4, and 6 h postinfection, the medium was removed; the monolayers were lysed with 0.1% saponin-PBS, and the cell lysates were serially diluted in PBS and plated on BHI agar plates supplemented with streptomycin (200 μg/mL). The plates were incubated at 37°C for 2 days, and colonies were enumerated.

### Immunization and aerosol challenge of mice with virulent M. tuberculosis Erdman strain.

Groups of BALB/c or C57BL/6 mice, 8/group, were primed with BCG intradermally (i.d.) or intranasally (i.n.). BCG-primed mice were either not boosted or boosted once or twice with 2 × 10^6^ CFU of a single recombinant attenuated L. monocytogenes vaccine or a combination of two recombinant attenuated L. monocytogenes vaccine candidates expressing multiple M. tuberculosis antigens and challenged 3 or 4 weeks later by exposure to aerosolized M. tuberculosis Erdman strain generated by a Collison type 6 jet nebulizer (CH Technologies USA, Waltham, MA) from 10 mL of M. tuberculosis bacterial suspension (1.6 × 10^5^ to 2.6 × 10^5^ CFU/mL) for 30 min followed by 5 min to allow for settling of bacteria. The challenge dose was verified by euthanizing two animals and assaying CFU in their entire lungs at day 1 postchallenge. The mice were euthanized at various times postchallenge, and the spleens and lungs were removed and assayed for bacillus burden as described by us previously (22).

### Immunization of mice and assay for intracellular cytokine staining of mouse spleen and lung cells.

To determine the immunogenicity of rLm5Ag(30) expressing the fusion protein of 5 M. tuberculosis antigens (23.5-10.4-ESAT6-CFP10-r30) as a booster vaccine, we immunized C57BL/6 and BALB/c mice, 4/group, subcutaneously (s.c.) with BCG at week 0; boosted them at weeks 14 and 18 with 2 × 10^6^ CFU of the L. monocytogenes vector or rLm5Ag(30); anesthetized, bled, and euthanized the mice at 6 days after the last immunization; prepared single-cell suspensions of spleen and lung cells; stimulated the single-cell suspensions without protein antigen (medium alone, negative control) or with a single M. tuberculosis protein, pool of multiple M. tuberculosis proteins, purified protein derivative (PPD), or PMA (positive control); and assayed T-cell immunity by intracellular cytokine staining (ICS) using methods that we published previously (22, 61) with modifications as described below.

We conducted ICS by using an eight-color flow cytometry panel to simultaneously analyze multiple cytokines at the single-cell level. Specifically, a single-cell suspension of 5 × 10^5^ lung cells per well or 1.0 × 10^6^ splenocytes per well was seeded in U-bottom 96-well plates and stimulated with medium alone (negative control), 5 μg/mL of recombinant proteins r30/Ag85B (our lab stock, isolated from recombinant Mycobacterium smegmatis); ESAT6/EsxA (BEI Resources); CFP10/EsxB (BEI Resources); TB10.4/EsxH (Aeras); 23.5/Mpt64 (BEI Resources); pool of 5 antigens (5Ag) comprising r30, ESAT6, CFP10, TB10.4, and 23.5, each at 2 μg/mL; or PPD (5 μg/mL) in the presence of anti-CD28 monoclonal antibody (clone 37.51) for a total of 6 h. Cells stimulated with PMA served as a positive control. Four hours prior to harvest, GolgiPlug (protein transport inhibitor containing brefeldin A) diluted in T-cell medium was added to all wells; PMA was additionally added to positive-control wells. Following *in vitro* stimulation, cells were harvested, washed with PBS, incubated with Live/Dead Fixable Near-IR cell stain (Invitrogen) for 10 min at room temperature to identify dead cells, and surface stained with antibodies against CD4 (clone RM4-5, conjugated with Brilliant Violet 510) and CD8 (clone 53-6.7, conjugated with Brilliant Violet 605). Cells were then fixed/permeabilized with Cytofix/Cytoperm (BD BioSciences) and stained for CD3 (clone 17A2, conjugated with Alexa Fluor 488 [AF488]), IFN-γ (clone XMG1.2, conjugated with Brilliant Violet 650), IL-2 (clone JES6-5H4, conjugated with PE), TNF-α (clone MP6-XT22, conjugated with PerCPCy5.5), and IL-17A (clone TC11-18H10.1, conjugated with Alexa Fluor 647). Note that due to the internalization of CD3 in responding CD4^+^ T cells, cells were stained for CD3 after fixing/permeabilization. The fluorochrome-conjugated antibodies were purchased from BioLegend. For flow cytometry analysis, a minimum of 100,000 lymphocytes per sample were acquired with an HTLSRII (BD) flow cytometer. The data were analyzed by using FlowJo software. The initial gating of total events included a lymphocyte gate, followed by selection for singlet cells and live CD3^+^ T cells (near-infrared AF488^+^); CD4^+^ and CD8^+^ T cells were identified by CD4^+^ (BV510^+^ BV605^−^) and CD8^+^ (BV605^+^ BV510^−^) expression, respectively. The gates for frequencies of antigen-specific IFN-γ-, IL-2-, TNF-α-, and IL-17A-producing CD4^+^ and CD8^+^ T cells were determined by using the unstimulated cells; Boolean combinations of the four intracellular cytokine gates were used to uniquely discriminate responding cells based on their frequency with respect to cytokine production. Each cytokine-positive cell was assigned to 1 of the 15 possible combinations. In some cases, background frequencies of CD4^+^ and CD8^+^ T cells producing cytokines without antigen stimulation were subtracted.

### Enzyme-linked immunosorbent assay for serum antibody specific for M. tuberculosis antigens in mouse sera.

Mouse sera were assayed for IgG antibodies specific to M. tuberculosis protein antigens of Ag85B, EsxA, EsxH, Mpt64, and PPD and to formalin-killed rLm5Ag(30) (FK-rLm5Ag). Briefly, high-binding 96-well plates (Costar) were coated with carbonate/bicarbonate buffer without antigen (control), M. tuberculosis protein antigens (1 μg/mL), or FK-rLm5Ag (1 × 10^8^/mL) overnight at 4°C, blocked in 3% bovine serum albumin (BSA) for 3 h at room temperature, washed 4 times, incubated with sera at 2-fold serial dilutions starting at 1:50 or with PBS overnight at 4°C, washed 4 times, and incubated for 90 min with alkaline phosphatase (AP)-conjugated goat anti-mouse IgG (Sigma, St. Louis, MO) at a dilution of 1:2,500 at ambient temperature. After the plates were washed 3 times, 100 μL of NPP (*p*-nitrophenylphosphate) substrate in diethanolamine buffer (phosphatase substrate kit; Bio-Rad, Hercules, CA) was added to each well and incubated for 20 min. The reaction was stopped by adding 100 μL of 0.1 N sodium hydroxide, and the solutions were read at 415 nm for absorbance. The endpoint antibody titer was calculated as the reciprocal of the highest immune serum dilution that gave a minimum difference of 0.05 optical density units when comparing the test (with antigen) and control (without antigen) wells.

### Statistical analyses.

Two-way analysis of variance (ANOVA) with Sidak’s multiple-comparison test was performed using GraphPad Prism v9.2.0 (San Diego, CA) to determine significance in comparisons of mean frequencies of lymphocytes, T cells and cytokine-producing CD4^+^ and CD8^+^ T cells, and serum IgG antibody titer between mice vaccinated with the L. monocytogenes vector and mice vaccinated with rLm5Ag (30). One-way ANOVA with Tukey’s multiple-comparison test and/or with Fisher’s least significant difference (LSD) test was performed using GraphPad Prism v9.2.0 (San Diego, CA) to determine significance in comparisons of means of log_10_ CFU in spleens and lungs among mice in vaccinated and control groups.
